# Divergent JAM-C Expression Accelerates Monocyte-Derived Cell Exit from Atherosclerotic Plaques

**DOI:** 10.1371/journal.pone.0159679

**Published:** 2016-07-21

**Authors:** Paul F. Bradfield, Arjun Menon, Marijana Miljkovic-Licina, Boris P. Lee, Nicolas Fischer, Richard J. Fish, Brenda Kwak, Edward A. Fisher, Beat A. Imhof

**Affiliations:** 1 Department of Pathology and Immunology, CMU, University of Geneva, 1211, rue Michel Servet 1, Geneva 4, Switzerland; 2 Division of Cardiology, New York University Langone Medical Center, New York, New York 10016, United States of America; 3 NovImmune S.A., 14 chemin des Aulx, 1228 Plan-les-Ouates, Geneva, Switzerland; 4 Department of Genetic Medicine and Development, CMU, University of Geneva, 1211, rue Michel Servet 1, Geneva, Switzerland; University Dresden, GERMANY

## Abstract

Atherosclerosis, caused in part by monocytes in plaques, continues to be a disease that afflicts the modern world. Whilst significant steps have been made in treating this chronic inflammatory disease, questions remain on how to prevent monocyte and macrophage accumulation in atherosclerotic plaques. Junctional Adhesion Molecule C (JAM-C) expressed by vascular endothelium directs monocyte transendothelial migration in a unidirectional manner leading to increased inflammation. Here we show that interfering with JAM-C allows reverse-transendothelial migration of monocyte-derived cells, opening the way back out of the inflamed environment. To study the role of JAM-C in plaque regression we used a mouse model of atherosclerosis, and tested the impact of vascular JAM-C expression levels on monocyte reverse transendothelial migration using human cells. Studies in-vitro under inflammatory conditions revealed that overexpression or gene silencing of JAM-C in human endothelium exposed to flow resulted in higher rates of monocyte reverse-transendothelial migration, similar to antibody blockade. We then transplanted atherosclerotic, plaque-containing aortic arches from hyperlipidemic ApoE^-/-^ mice into wild-type normolipidemic recipient mice. JAM-C blockade in the recipients induced greater emigration of monocyte-derived cells and further diminished the size of atherosclerotic plaques. Our findings have shown that JAM-C forms a one-way vascular barrier for leukocyte transendothelial migration only when present at homeostatic copy numbers. We have also shown that blocking JAM-C can reduce the number of atherogenic monocytes/macrophages in plaques by emigration, providing a novel therapeutic strategy for chronic inflammatory pathologies.

## Introduction

In atherosclerosis, arterial plaque formation is initially triggered by a continued accumulation of normal and modified lipoproteins in the subendothelial layer. It is driven by a chronic and maladaptive inflammatory response, wherein tissue resident macrophages derived from monocytes become engorged with cholesterol and persist in the lesion instead of being cleared through emigration or otherwise [1, 2]. Importantly, plaques have a relatively high composition of these inflammatory cholesterol-loaded macrophages or foam cells, commonly identified as CD68^+^ or Oil-red O positive [3]. As recently reviewed, we and others have shown under conditions of profound lipid lowering in hypercholesterolemic mouse models, the pro-inflammatory state of the plaque can be resolved rapidly in some of these models by the emigration of monocyte-derived cells [1].

Recruitment of monocytes to sites of tissue injury/inflammation and atherosclerotic plaques, is tightly regulated by a series of events involving interactions between adhesion molecules. Monocyte capture and adhesion is followed rapidly by transendothelial migration (TEM) into the tissue, through intercellular endothelial junctions [4, 5]. A major component of the endothelial barrier is the tight junction, which is composed primarily of occludin, claudins and junctional adhesion molecules (JAMs) [6]. JAMs are members of the immunoglobulin superfamily and contain 2 Immunoglobulin (Ig) domains [4]. The JAM family is composed of the 3 classical members (JAM-A, JAM-B and JAM-C) with a short cytoplasmic tail and 4 non-classical molecules with a long cytoplasmic tail [6–10].

Vascular JAM-C expression has been shown to play a critical role in inflammatory disease and metastasis [11–21]. It has been recently observed that following TEM, neutrophils and monocytes can re-enter the circulation [4, 22–25]. This process, called reverse-transendothelial migration (rTEM] is regulated by vascular JAM-C expression [4] and it has been proposed as an inflammatory resolution mechanism wherein transmigrated leukocytes exit into the circulation [26, 27]. However, leukocytes that have undergone rTEM remain in an activated state, and can result in cumulative inflammation at distal sites [22, 25, 27]. In chronic inflammation, expression of JAM-C is increased in diseases such as arthritis and atherosclerosis [11, 12, 17]. More acute models have exhibited a reduced expression and disrupted distribution profiles [22]. Furthermore, proteolytic cleavage of endothelial JAM-C is decisive in dissemination of inflammation [19, 27]. However, it is important to be aware of potential exceptions to this apparent trend. Upregulation of JAM-C in a transgenic mouse has been reported to exacerbate the disease process in an acute inflammatory model of pancreatitis [14].

In the present study, we have examined the effect of blockade of JAM-C in a mouse model of atherosclerotic plaque regression, as well as increased and reduced endothelial JAM-C expression on monocyte rTEM in-vitro. Blocking JAM-C function through injection of antibody (Ab) was found to cause a greater reduction in the critical plaque area as well as in the relative inflammatory cell content of these lesions following the exposure of advanced plaques to a normolipidemic environment. This occurred without significant changes to either circulating leukocyte subpopulations or their recruitment into the plaques, consistent with our previous findings in-vitro of no effect of JAM-C blockade on the primary-TEM of monocytes through endothelial cells [4]. Notably, we directly demonstrate that the decrease in the content of the monocyte-derived (CD68^+^) cells is through their increased egress from the plaques, which is consistent with the effects of JAM-C blockade on rTEM in-vitro.

## Materials and Methods

### General reagents

Unless otherwise noted, reagents were purchased from Sigma-Aldrich Chemie GMBH (Buchs, Switzerland). Antibodies (Abs) used for flow cytometry and/or immunofluorescence were anti- Vascular endothelial (VE)-Cadherin (BD Biosciences, Allschwil, Switzerland) and Alexafluor 488 conjugated anti- Platelet/endothelial cell adhesion molecule-1 (PECAM-1)/CD31 (Biolegend, USA), anti-JAM-B, anti-ICAM-1 (RND Systems, Abingdon, UK), anti-zonula occludens-1 (ZO-1), anti-Occludin-1 and anti-claudin-5 (Life Technologies Europe BV, Zug, Switzerland). Rabbit serum prior to immunization was used as a control (day 0) for labeling with polyclonal to human JAM-C (day 74) (Covalab, Lyon, France). Alexafluor 488 conjugated goat anti-rabbit IgG (Invitrogen) and Fluorescein isothiocyanate (FITC) conjugated goat anti-mouse IgG (Jackson Immunoresearch, USA). FITC conjugated Anti-CD62L, and anti-CD14 (BD Biosciences). For histology and flow cytometry on Human umbilical vein endothelial cells (HUVECs), JAM-C studies were conducted using antibody 225.3, or a mouse anti-human IgG1isotype control (both produced in-house). For flow cytometry studies on HUVECs transfected with Small interfering RNA (siRNA), an anti-JAM-C antibody H33 conjugated to Alexa-488 was used. Labelling was done with a goat anti-human FITC or PE-conjugated secondary antibody (Jackson Immunoresearch, USA).

### Carotid artery preparation and JAM-C visualization

Carotid arteries were isolated from young (6-wks) and old (24-wks) ApoE^-/-^ mice were perfused with Cryo-OCT compound (Fisher Scientific, Wohlen, Switzerland) and snap frozen in liquid nitrogen. Samples were cut into 10 μm sections and fixed in 4% paraformaldehyde. Samples were labelled with Alexafluor 488 conjugated anti-PECAM-1/CD31 and purified rabbit polyclonal anti-JAM-C followed by DyLight594 conjugated anti-rabbit secondary. Nuclear staining was performed using 4',6-diamidino-2-phenylindole (DAPI) and samples were visualized with confocal microscopy (LSM510; Carl Zeiss, Feldbach, Switzerland). JAM-C expression was evaluated using ImageJ software with pixel intensity being the readout of relative expression level. Fiji (ImageJ) software was used to quantify endothelial JAM-C expression on stained sections of carotid arteries. Measurements were conducted on three images from two independent experiments for each of two conditions (10-wks vs 6-mths). The maximal pixel intensity was measured for 6–9 region-of-interests (ROI) for each image, and the average maximal pixel intensity per condition was calculated.

### Endothelial cell preparation and culture

Human umbilical vein endothelial cells (HUVEC) were isolated by collagenase treatment of umbilical veins as previously described [28–31] and maintained in M199 containing 10% fetal calf serum (FCS), 15 ug/ml endothelial cell growth supplement (Upstate Biotechnology, Lake Placid, NY), 100 ug/ml heparin, 50 uM hydrocortisone and 10 ug/ml vitamin C. Umbilical cords were collected within 12-hrs of delivery for endothelial cell isolation. Briefly, the cord vein was cleared by perfusing with PBS, followed by incubation with collagenase (Invitrogen, 1 mg/ml) in PBS for 15-mins at 37°C. The vein was then perfused with PBS to remove the cells and centrifuged at 200 x g for 5 minutes. The cell pellet was then resuspended in complete M199 medium and transfered to culture. Cells were cultured up to passage 5.

### Transfection of JAM-C siRNAs

HUVECs were transfected with 300 nM human JAM-C siRNAs1 and 2 (JAM3-HSS130321 and JAM3-HSS130322 respectively; Stealth; Invitrogen, Carlsbad, CA) using the Amaxa Nucleofector (Life Technologies) and cultured for 24-48-hrs before experimentation. Inhibition of JAM-C expression in HUVECs after transfection was compared with transfection using control siRNAs: siRNA non-homologous to any known human gene (ctrl siRNA) or mock (buffer only). Expression of target and reference genes were analyzed by real-time polymerase chain reaction (qPCR). The sequence for JAM-C-specific primers were as follows: Forward 5'- aag aac cca ggg aaa cca gat gga-3'; Reverse 5'- tcg ctg cct tga cag gag ttt cta-3’. The values were normalized to the expression levels of human beta-actin, beta-tubulin, and Glyceraldehyde-3-phosphate dehydrogenase (GAPDH), according to the GeNorm method [32]. Analysis for JAM-C expression was performed by qPCR and flow cytometry. HUVECs were selected for experimentation if silencing induced 70% decrease or more in human JAM-C expression [4].

### Upregulation of JAM-C expression

Construction of lentivirus JAM-C-Enhanced green fluorescent protein (EGFP) (LV-JAM-C-EGFP) as well as control EGFP lentivirus (LV-EGFP) vectors has been previously described [23, 33]. EGFP was inserted into the hinge region of JAM-C between the membrane proximal C2 domain and the transmembrane region. The JAM-C-EGFP cDNA was transferred to a lentivirus expression vector, and high-titer virus stocks were produced. These stocks were titrated by a previously described protocol for expression in HUVEC [34]. Expression of JAM-C-EGFP in 70–80% of endothelial cells was observed up to 5-days after transfection, which resulted in a stable increase in JAM-C-EGFP expression of 1.5–3 fold compared to controls [23]. For all flow assay experiments, the control HUVEC cells were transfected with the EGFP LV control to yield 70–80% transfection. The high titre of JAM-C-EGFP was generated by adding 10-times the standard concentration, which typically generated levels that were 6- to 7-times higher than JAM-C- Wild-type (WT) levels.

### Immunostaining of HUVEC monolayers

Slides containing HUVEC monolayers were fixed in methanol (−20°C) for 5-mins and washed in Phosphate buffered saline (PBS) containing 0.5% BSA wash buffer. Monolayers expressing JAM-C-EGFP were fixed for 20-30-mins in methanol to inactivate EGFP or with 4% paraformaldehyde to maintain EGFP activity. Preincubation with human serum was conducted before immunostaining. Slides were mounted in Mowiol/DABCO for confocal microscopy (LSM510; Carl Zeiss, Feldbach, Switzerland).

### Monocyte isolation

PBMCs were first isolated from blood of healthy donors followed by monocyte purification using a monocyte isolation kit (Miltenyi Biotec, Bergisch Gladbach, Germany). Purity and activity of monocytes were controlled by flow cytometry by labelling with anti-CD14 and anti-CD62L Abs (data not shown). Only populations of 90% or more double positive cells were used.

### Shear flow assays

HUVECs were cultured at 4 to 5 × 10^5^ cells/slide as previously described [4] for 2- to 3-days and treated for 4-hrs with Tissue Necrosis Factor alpha (TNF-alpha) (500 U/ml). The slides were attached to a flow chamber at 37°C (CAF10; Immunetics, Cambridge, MA) and flow was generated over the HUVEC monolayer by perfusing wash buffer (M199 with 0.5% BSA) or a monocyte suspension (2.5 × 10^6^ cells/ml) using a calibrated pump (74 900; Cole-Parmer, Vernon Hills, IL). The flow rate was representative of shear rates in small venules/capillaries (0.05 Pa/0.5 Dynes). Flow assays were conducted as described in a previous study [4]. Observations made using phase-contrast microscopy (Model: Axiovert 35; Objective: 20×/0.3 NA air; Carl Zeiss) were recorded using a high-resolution camera (D70; Nikon, Zurich, Switzerland). Individual images were recorded every 30 seconds with Nikon capture software (v4.2) and compiled into movie sequences using Adobe Photoshop (v7.0) and Image J (v1.33u), allowing analysis of individual monocytes over large areas. Concentrations were maintained at 50 ug/ml for the monoclonal anti-murine JAM-C H33/H36 Abs due to the low affinity and 10 ug/ml for the high affinity anti-human JAM-C 225.3. Preincubation of HUVECs was performed 20-mins prior to addition of monocytes. Monocytes were perfused over activated HUVECs for 5-mins followed by 20- to 60-mins wash buffer. For individual cell tracking, the phase appearance of each monocyte was marked at 1-min intervals. RTEM was determined by tracking individual transmigrated monocytes, and was defined as migration in the abluminal-to-luminal direction for 1-min or more. Monocytes typically showed a robust pattern of migration between endothelial compartments, consistent with our previous study [4]. Multi-directional migratory behaviour; consistent with hesitancy in the abluminal compartment as described in a previous study [22], was observed in less than 5% of the monocytes counted across all experimental groups (data not shown). TEM and rTEM events are presented as a percentage of total monocytes captured from flow per unit field. Physiological flow was applied at all times during monocyte:HUVEC coculture where percentage TEM of adherent monocytes typically exceeded 90% in this and a previous study [4]. All flow assays were performed using TNF-alpha activated HUVECs, as no monocytes were captured from flow under non-inflammatory conditions (Data not shown). All experiments were carried out using triplicate fields and presented as a mean value (±SEM). The time of abluminal occupation was calculated by subtracting the timepoint of a rTEM event (as defined previously) from the time of completed primary TEM for each individual monocyte.

### Biacore surface plasmon resonance analysis

Interactions studies were performed on a Biacore 2000.The human soluble JAM-C (up to the QEMEV sequence) protein flagged at the C terminus was produced in BOSC cells and purified through an anti flag column. The protein was immobilized at a concentration of 1 ug/ ml on a M5 sensor chip using the amine coupling kit (NHS-EDC) provided by the Biacore Supplier to the level of 155 resonance units. The background signal from a reference channel without soluble JAM-C was automatically subtracted. Protein G purified Abs (50 ul) were injected at a flow rate of 20-ul/min, in the provided running buffer at different concentrations: 100, 50, 10, 5 and 1 ug/ ml.

### Animals, Aortic Transplantation and Leukocyte Trafficking in-vivo

All procedures described were approved by the Animal Use and Care Committee at the NYU School of Medicine and the Institutional Animal Care and Use Committee (IACUC). The model of aortic arch transplantation has been previously detailed [35–37]. Briefly, the aortic arch is humanely excised from a “donor” mouse and is implanted into the abdominal aorta of a “recipient” mouse by end-to-side anastomoses, with the intervening abdominal aortic segment tied off to divert all of the blood flow through the arch segment.

ApoE^-/-^ mice on a C57BL/6 background (all mice detailed were similarly from this background) were weaned at 3-4-wks old onto a high-fat Western Diet (WD, 21% [wt/wt] fat, 0.15% cholesterol, Research Diets). Mice were maintained on WD for 12-wks, after which a subset was used for baseline measurements (BL), and the remainder were used for transplantation procedures. 48-hrs prior to sacrifice, all donor mice were injected with 5-ethynyl-2’-deoxyuridine (EdU) (2 mg/kg, i.p.) in order to allow EdU incorporation into DNA during active monocyte precursor proliferation in the bone marrow. The released, labeled monocytes will then enter various peripheral tissues, including the atherosclerotic plaques. After transfer of the aortic arches containing the labeled cells in the plaques, if some of them emigrate in the regression (normal lipid environment), the difference between the number of baseline lesional EdU^+^ (i.e., labeled) cells and the recipient transplanted arch lesional EdU^+^ cells will reflect leukocyte egress in each group [38].

For quantification of EdU^+^ cell content of the plaques, frozen aortic arch segment sections were allowed to thaw to room temperature, and then fixed with 4% paraformaldehyde in PBS for 1-hr. After washing twice with a 3% BSA in PBS serum solution, the sections were permeabilized with 0.5% Triton X-100 in PBS for 1-hr. The sections were again washed twice with 3% BSA in PBS and then incubated with a Click-iT reaction cocktail containing Click-iT reaction buffer (Invitrogen), CuSO4, Alexa Fluor 647 Azide, and reaction buffer additive for 1-hr while protected from light. The sections were washed once more with 3% BSA in PBS. For subsequent nuclear staining, sections were washed once with PBS and then incubated with 5ug/ml DAPI solution for 15-mins. Finally the slides were washed twice with PBS and coverslipped with Prolong Gold Antifade Reagent (Life Technologies). Cells were imaged using confocal microscopy, with EdU^+^ cells identified by Alexa Fluor 647. Quantification of EdU^+^ cells/section was performed by counting AF647^+^ cells in the indexed fashion similar to morphometrics and bead assay. All age-matched recipient mice were WT on the C57BL/6 background. The recipient groups consisted of: 1) PBS treated controls (WT), 2) i.v. injected with 8mg/kg monoclonal JAM-C at 24-hrs prior to transplantation and 24 and 48-hrs afterwards (JAM-C), or 3) Mice injected similarly with a JAM-C isotype control (Biolegend IgG2A K, IgG). Recipients were maintained on chow diet following surgery and were sacrificed 4-days post-transplantation. 48-hrs before recipient sacrifice, all recipients were injected with fluorescent latex beads diluted in PBS 1:4 (Polysciences Inc.), which are taken up by circulating monocytes [39]. Since they do not degrade over time, the number of beads in the plaque gives a relative measure of new monocyte entry or “recruitment” in the plaque and can be compared across groups to potentially highlight different dynamics of leukocyte recruitment. Beads per section were counted by imaging FITC+ cells, and this was done in an indexed fashion similar to morphometric analysis (2 measurements per slide, with one from every index). To correct for potential bead-labeling differences in the monocyte population between mice, labeling efficiency was measured 24-hrs post-injection by drawing blood and measuring the percentage of bead-positive monocytes in recipients using flow cytometry (see below and [38, 39]). In all instances, mouse housing and husbandry was conducted according to IACUC regulations. The mice were monitored on a daily basis; none became severely ill or died at anytime prior to experimental endpoints. All mice were sacrificed using a CO_2_ euthanasia.

### Lesion Morphometrics and Assessment

Following perfusion with 10% sucrose, baseline aortic arches and transplanted arch grafts were removed, embedded in OCT, and frozen. Serial sections of 6 μm were obtained and mounted on glass slides. For lesion immunohistochemistry (IHC), sections were fixed in 100% acetone and stained for CD68^+^ using primary rat anti-mouse CD68 (Serotec), followed by biotinylated anti-rat IgG secondary, and visualization using a Vectastain ABC kit (Vector Laboratories). Briefly, sections were blocked with 4% rabbit serum, stained for 1-hr with primary CD68, incubated 20-mins with biotinylated IgG, treated for 5-mins with alkaline phosphatase, and treated 8-10-mins with Vector Red substrate in Tris-HCl buffer. Vector red staining of sections was stopped with ddH20 and subsequently underwent a standard hematoxylin/bluing counterstaining, dehydrated to xylene and mounted with coverslips [36, 40]. Morphometrics were analyzed using ImageProPlus 7 (Micro Optical Solutions) on bright-field images of stained sections (10X magnification), with at least 2 measurements per slide. To account for changes in the axial length of the arch, 4–7 indexes of sections were taken per arch, and one slide from each index was analyzed, giving a representation of the entire arch. This indexing technique was similarly applied to bead-labeling and EdU-labeling measurements. Total plaque area, plaque % of lumen, CD68^+^ total area, and CD68^+^ % of lesion area were used as four summary parameters of the lesion morphometrics.

### Flow Cytometry

#### Blood Leukocytes

Leukocytes were identified from collected blood using previously established methods [41]. Briefly, blood was drawn from tails with heparinized and EDTA-coated capillaries or from the heart through cardiac puncture at sacrifice and collected in EDTA containing tubes to prevent coagulation. Blood samples were then subjected to erythrocyte lysis buffer (Pharm Lyse, BD) to lyse red blood cells (RBCs) for 30-mins, pelleted, and white blood cells were resuspended in flow cytometry buffer (0.5%BSA, 5mM EDTA in HBSS) at 4C. Samples were pelleted and washed again before staining with a panel of Abs. Leukocytes were identified by CD45^hi^ staining (CD45: Phycoerythrin (PE)-Cy7), and from this monocyte populations were characterized as by CD115 and Ly6-C/G (CD115: PE, Ly6-C/G: APC, all eBioscience), such that Ly6Chi monocytes were CD115^hi^Ly6-C/G^hi^, Ly6C^lo^ monocytes were CD115^hi^Ly6-C/G^lo^, and neutrophils were CD115^lo^Ly6-C/G^lo^.

For bead labeling normalization, the % of FITC-positive monocytes was analyzed 24-hrs after injection to determine relative degree of labeling when comparing across groups. Other CD45^+^ populations were similarly analyzed for FITC to check for non-specific labeling which might obscure results. Flow cytometry was performed using a LSRII analyzer (BD), and analysis was performed using FlowJo X software (Tree Star).

#### White Blood Cell Count

Total white blood cell count from collected mouse blood was performed using a hematology cell counter (Oxford Science Inc.). At sacrifice, relative populations were calculated as a percentage of total CD45, CD45+/CD115+/ly6c^hi^, and CD45+/CD115+/ly6c^low^ groups by flow cytometry from a terminal bleed with a volume of 200 ul. A volume of 50 ul of blood was also taken for hemacytometer measurements, allowing the total number of white blood cells (CD45+) to be calculated. Using these measurements the percentage of each population was calculated (CD45+ number * % of each population).

#### Statistical Analysis

Data presented is expressed as mean±standard error measurement (SEM) or as Median values. For all comparative analysis of morphometrics, leukocyte populations, and atherosclerosis, 1-way ANOVA was used with Tukey-Kramer post-hoc analysis when appropriate. All other analysis was conducted using the Student unpaired T-test unless otherwise stated. Significance was marked as follows: NS = Not Significant (P>0.05), * = p<0.05, ** = p<0.01, *** = p<0.001 and **** = p<0.0001.

## Results

### Ab blockade of JAM-C promotes inflammation resolution in regressing atherosclerotic plaques

In order to investigate the effect of JAM-C blockade on monocyte/macrophage trafficking in atherosclerosis, we used a mouse aortic transplantation model. Atherosclerotic plaques were induced by feeding ApoE-/- “donor” mice a Western-type diet (WD) for 12-weeks (wks). It has been shown that transplantation of the atherosclerotic aortic arch into a normolipidemic recipient (wild type; WT) mouse resulted in dramatic plaque regression [35, 36], in part, at least, attributable to emigration of monocytes/macrophages (CD68^+^ cells) from the plaques.

**Fig 1:**
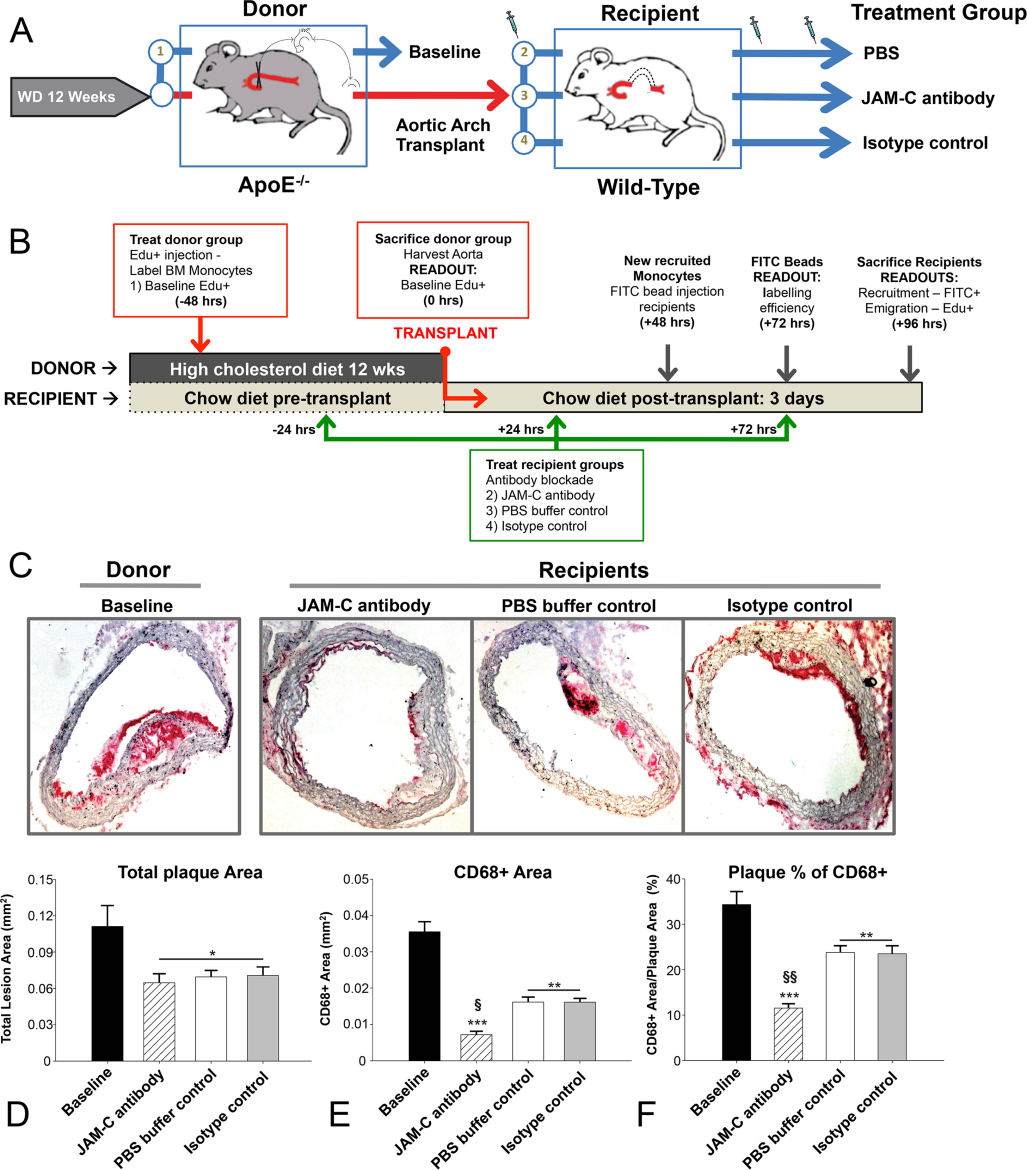
Ab blockade of JAM-C reduces plaques in a transplantation model of atherosclerosis. (A) Scheme illustrating how atherosclerotic arches are generated in ApoE^-/-^ donor mice preceding transplantation into treated or untreated normolipidemic WT mice. (B) Timeline of treatment and monocyte tracing in the aortic arches to establish recruitment and emigration profiles. Aortic arches were harvested from donor ApoE^-/-^ mice (baseline) and transplanted or not into WT recipient mice treated with PBS buffer control, anti-JAM-C (JAM-C antibody) or an isotype control antibody (isotype control). Tissue sections were stained for CD68^+^ and visualized with Vector Red substrate. (C) Representative images from each group are shown. Morphometrics were analyzed using the ImageProPlus7 program with at least 2 measured areas per slide. To account for changes in the axial length of the arch, 4–7 indexes of sections were taken per arch, and 1 slide from each index was analyzed so that at least 4 slides were analyzed for each aortic arch vessel, and the mean value was used as the summary parameter. (D) Total plaque area, (E) CD68^+^ area, and (F) CD68^+^ as a percentage of the total plaque area were used as parameters of the lesion morphometrics. Data are presented as the mean ±SEM (N = 6). P values marked *—*** were calculated compared to baseline measurements. Both § and §§ = p<0.01 were compared to WT and IgG recipients.

Therefore we transplanted the atherosclerotic aortic arch into WT recipient mice, and these were sacrificed 4-days later (Fig 1A and 1B). Immunostaining of serial sections for CD68^+^ was used to determine cells that were presumably monocytes and inflammatory cholesterol-loaded macrophage foam cells, and quantification of atherosclerosis through CD68^+^ content has been extensively used in the past in this regard (Fig 1C, Representative CD68^+^ images per group). Although neutrophils can also express CD68, there was only background staining for Ly6G (a neutrophil marker) in all groups. Some minor populations of cholesterol-loaded smooth muscle cells can also show CD68 expression, however their speed of migration is relatively low compared to monocytes, and there is no evidence of massive short-term immigration or emigration from plaques (see below) [42, 43]. Consistent with previous studies [36, 37], the total plaque area (the intimal area occluding the lumen) was significantly less in the PBS buffer-control recipient group than in the pre-transplant baseline (BL) group (Fig 1D; 0.07 ±0.005 mm^2^ compared to 0.11 ±0.017 mm^2^, P<0.05). This change in total area was attributed to a decrease in the plaque inflammatory cell content, taken as the area of CD68^+^ staining (Fig 1E), which was 0.016 ±0.001 mm^2^ in PBS buffer-control recipients vs. 0.036 ±0.003 mm^2^ in Basline (BL) donors (P<0.001). We measured the relative monocyte/macrophage content in the plaque (CD68^+^ area/total plaque area, %, Fig 1F) and found, as predicted by the absolute areas, that a significantly lower inflammatory content was found in PBS buffer-control plaques: 23.8 ±1.5% CD68^+^, while BL were 34.3 ±2.9% (P<0.01).

Interestingly, recipient mice that were treated with a blocking anti-JAM-C monoclonal antibody (H33) 24-hrs pre-transplantation, and 24- and 72-hrs post-transplantation showed significantly greater regression of the inflammatory lesion (Fig 1C). While the total lesion area in these JAM-C antibody treated recipients was similar to the PBS buffer-control recipient group (Fig 1D), the monocyte/macrophage content area was significantly reduced (Fig 1E. 0.007 ±0.001 mm^2^ compared to 0.016 ±0.001 mm^2^, P<0.01). The percentage of the inflammatory macrophage area of the total lesion of the anti-JAM-C antibody treated recipients was also reduced compared to the PBS buffer-control recipients (Fig 1F. JAM-C 11.5 ±0.9% vs. WT 23.8 ±1.5%, P<0.001). These trends were similar when comparing the anti-JAM-C treated recipients to mice treated with isotype control antibody. Neither the anti-JAM-C treatment nor the isotype control antibody affected the plasma lipid levels (data not shown).

As we have observed previously [41, 44], reduction in macrophage content in atherosclerotic aortas after transplantation into WT recipients occurred independently of the total plaque area. This has generally been attributed to the remodelling of the plaque either through increased collagen content that counterbalances a reduced recruitment and retention of circulating monocytes, or the increased emigration of plaque macrophages. Taken together, these data suggest that antibody blockade of JAM-C improves regression of inflammatory monocyte/macrophages in plaques as determined through standard morphometrics.

### Pre-treatment of JAM-C Ab does not affect blood leukocyte populations in normolipidemic recipient mice

**Fig 2:**
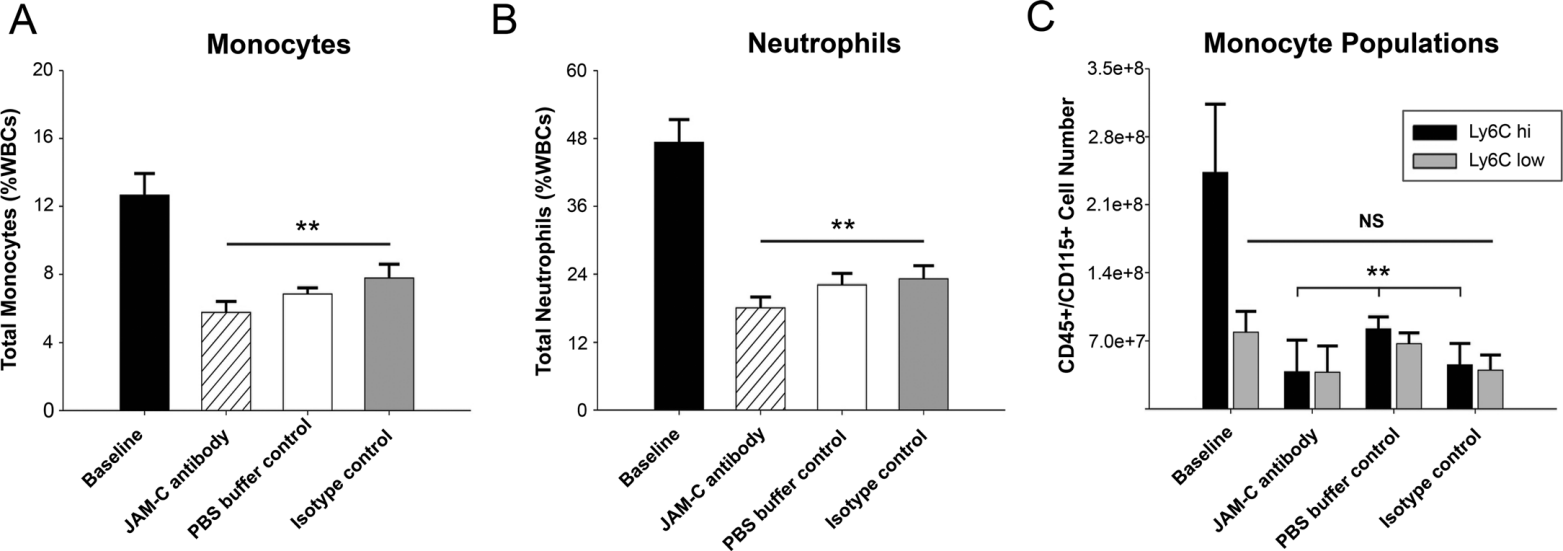
Flow cytometry data from blood monocytes. WBCs were counted by a hemocytometer after red blood cell lysis and stained with leukocyte markers from donor mice (Baseline) and recipient mice groups (JAM-C antibody, PBS buffer-control or an isotype control). (A) Total monocytes were gated for CD45^+^/CD115^hi^ and expressed as % of total WBCs. (B) Total neutrophils were defined as CD45^+^/CD115^lo^/Ly6C^hi^ and expressed as % of WBCs. Data are presented as the mean ±SEM (N = 6). (C) Monocytes were analyzed for Ly6C expression to differentiate Ly6C^hi^ and Ly6C^lo^ populations. NS = not significant. P values marked ** were calculated compared to baseline measurements. Both § and §§ = p<0.01 compared to PBS buffer-control and isotype control recipients.

To test for the possibility that interference with JAM-C and monocyte TEM may elicit a response in circulating leukocytes, we evaluated leukocyte populations from mouse whole blood at sacrifice. As expected, hyperlipidemic baseline mice exhibited monocytosis and neutrophilia [45], and had significantly higher levels of total monocytes (12.7 ±1.3%, P<0.01 to all recipients) and neutrophils (47.0 ±4.0%, P<0.001 all recipients) compared to all normolipidemic recipient subsets (Fig 2A and 2B) [38, 39, 45, 46]. No differences in monocyte or neutrophil profiles were observed across anti-JAM-C antibody, isotype control, or PBS buffer control recipients.

In the CD45^+^/CD115^+^ monocyte pool, Ly6C^hi^ and Ly6C^lo^ cell numbers were evaluated at sacrifice (Fig 2C, Ly6C^hi^ and Ly6C^lo^, cell number). Baseline ApoE^-/-^ mice after WD had a significantly increased number of Ly6C^hi^ monocytes (2.4E8 ±0.7E8, P<0.05 to all recipients) as expected from their hypercholesterolemia [39], but no differences were observed in Ly6C^hi^ monocytes across the normolipidemic recipient WT groups. No differences between any groups were present in Ly6C^lo^ monocytes. These data suggest that anti-JAM-C antibody treatment does not affect circulating leukocytes in a manner that would confound the interpretation of the results.

### Plaque-associated endothelium from ApoE^-/-^ mice shows increased JAM-C at intercellular junctions

**Fig 3:**
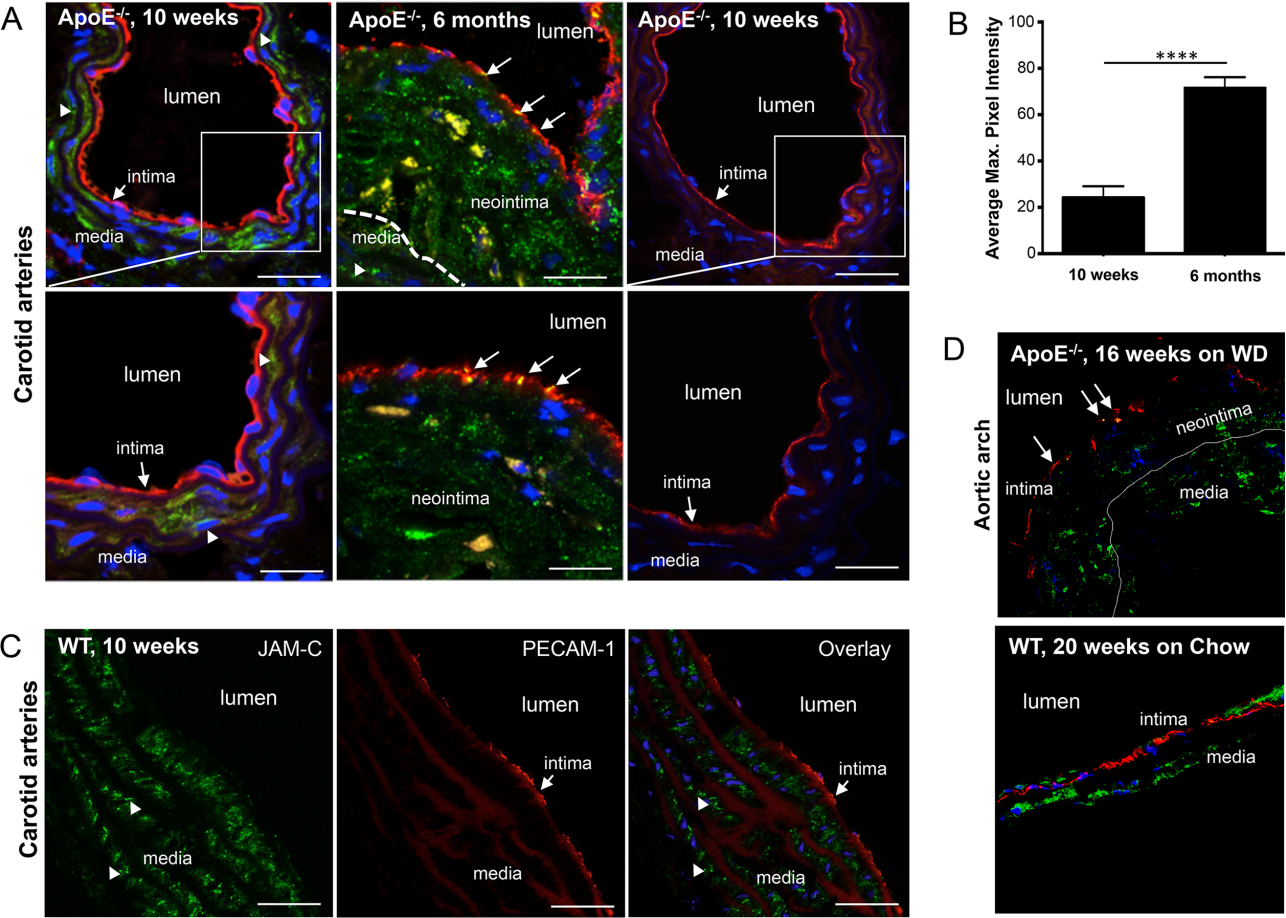
JAM-C expression in lesion-free and atherosclerotic carotid arteries. **(A)** Carotid artery sections of 10-wk old, lesion-free (left panels) and 6-mths old atherosclerotic ApoE^-/-^ mice (middle panels) stained for JAM-C and PECAM-1 expression. JAM-C (green) and PECAM-1 (red) immunostaining of carotid arteries shows JAM-C expression on smooth muscle cells (arrowheads) and endothelial cells of carotid arteries of 6-mths old, atherosclerotic ApoE^-/-^ mice (middle panels, arrows). No JAM-C staining was observed when an isotype control antibody was used (right panels). DAPI staining was used for nuclear counterstain (blue). Bars correspond to 100-um (upper panels); 200-um (lower panels). (B) Quantification of endothelial JAM-C expression of healthy (10 wks) and atherosclerotic carotid arteries (6 mths). Pixel intensity of endothelial associated JAM-C was measured using Fiji (ImageJ) software. Data presented as mean values ±SEM. (C) JAM-C expression in wild-type carotid arteries. Carotid artery sections of 10-wks old wild-type C57BL6/J mice stained for JAM-C and PECAM-1 expression. JAM-C (green) and PECAM-1 (red) immunostaining of carotid arteries shows JAM-C expression only on smooth muscle cells (arrowheads) and not endothelial cells (intima, arrows). Bars correspond to 100-um. (D) JAM-C expression in lesion-free and atherosclerotic aortic arches. Aortic arch sections of 16-wks old, atherosclerotic ApoE^-/-^ mice fed on Western-type diet (WD, left panel) and 20-wks old, lesion-free WT mice fed on normal chow diet (Chow, right panel) stained for JAM-C (green) and PECAM-1 (red) expression. JAM-C expression on endothelial cells of neointima in aortic arches of atherosclerotic ApoE^-/-^ mice (left panel, arrows). DAPI staining was used for nuclear counterstain (blue). Bars correspond to 100-μm. Images are representative of multiple stains done in at least two separate mice.

Having established that JAM-C blockade led to a greater reduction in plaque CD68^+^ cells in our mouse model of atherosclerosis regression, we investigated the distribution of JAM-C within the atherosclerotic plaques. Previous studies from our group and others showed that JAM-C localization at the arterial endothelium could be observed during chronic inflammation [11, 17]. To provide evidence of JAM-C expression and localization in the atherosclerotic carotid arteries, we carried out immunostaining on samples from both 10-week old plaque-free ApoE^-/-^ mice and 6-month (mth) old ApoE^-/-^ mice that developed spontaneous atherosclerotic lesions. As expected, we could not detect JAM-C expression in the arterial endothelium of plaque-free mice (Fig 3A, left panels). Similarly, WT mice of the same age showed no JAM-C expression in the arterial endothelium (Data not shown). However, the spontaneous atherosclerotic plaques in 6-mth old ApoE^-/-^ mice displayed JAM-C localization in the intercellular junctions of endothelial cells overlying atherosclerotic lesions (as determined by platelet endothelial adhesion molecule-1 (PECAM-1) expression) (Fig 3A, middle panels). No staining was observed with the relevant isotype control antibody (Fig 3A, right panels). We then quantified the level of JAM-C expression by measuring the pixel intensity on stained sections of carotid arteries isolated from 10-wk and 6-mth old mice (Fig 3B). These readings confirmed that there was a marked increase in JAM-C expression on the carotid arteries of mice with atherosclerotic plaques. JAM-C was also detected in the smooth muscle cells of the media on both carotid arteries and aortic arches in ApoE^-/-^ and WT mice (Fig 3A left panels and 3C, arrowheads). Interestingly JAM-C/PECAM-1 co-expression can also be seen on smaller microvessels in close proximity to the larger carotid artery (Fig 3A middle panel). Additionally, we carried out immunostaining of aortic arch samples from both 16-week old ApoE^-/-^ mice fed on WD that develop atherosclerotic lesions and 20-week old plaque-free WT mice fed on normal chow diet. JAM-C expression was observed exclusively in the endothelial junctions of aortic arches of atherosclerotic ApoE^-/-^ mice compared to the endothelium of WT mice (Fig 3D, ApoE-/- top panel and WT bottom). These data show that endothelial JAM-C expression is induced in intercellular endothelial junctions during atherosclerotic lesion progression in ApoE^-/-^ mice.

### JAM-C blockade and increased JAM-C expression enhance monocyte rTEM in-vitro

**Fig 4:**
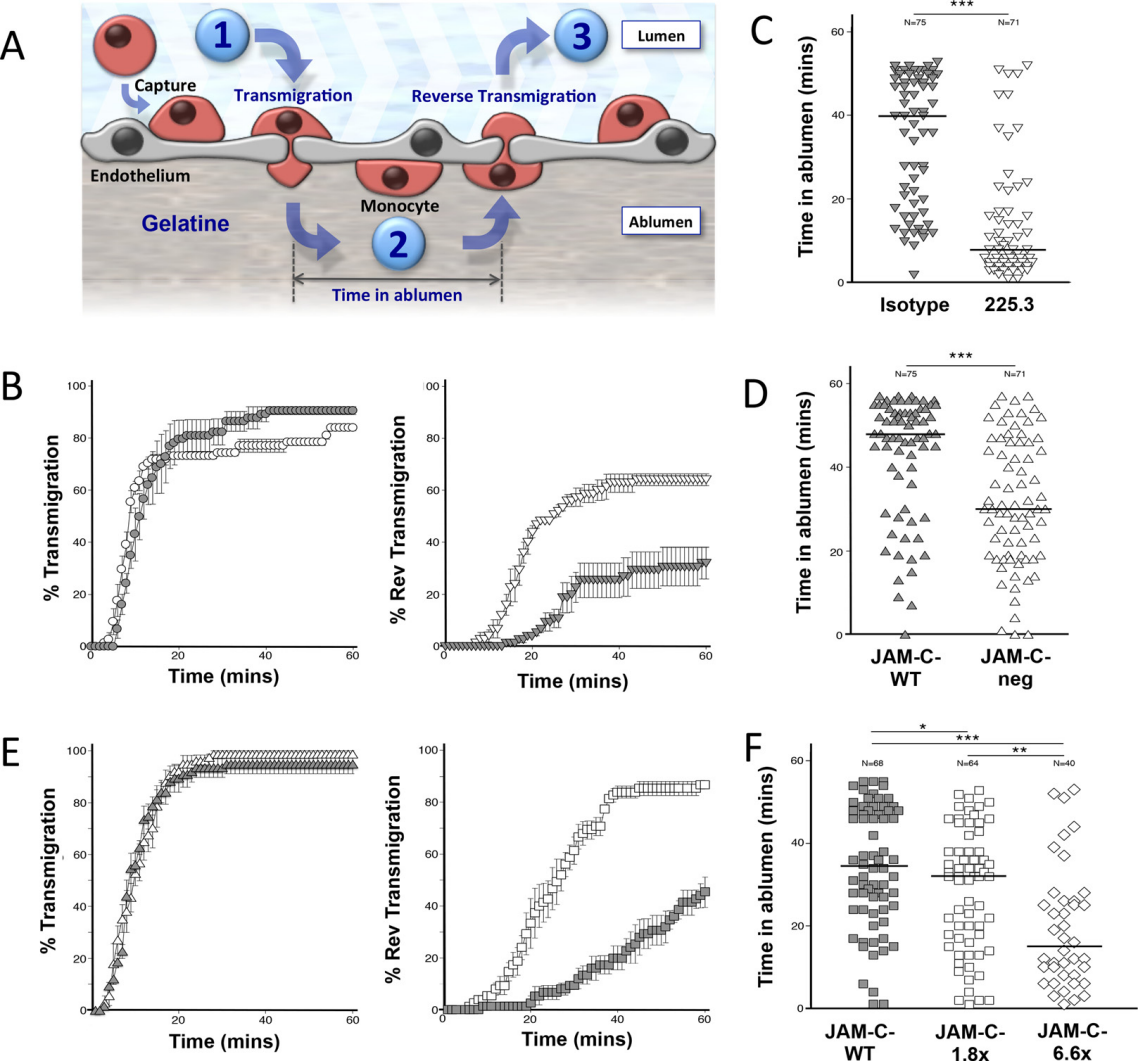
Trafficking profiles of monocytes on activated HUVECs in a flow assay system. Scheme of monocyte adhesion, TEM and rTEM. (A) Monocytes captured from flow adhere to HUVEC luminal surfaces before transmigrating into the abluminal compartment (Step-1). The period of time occupied by the monocyte can be measured (Step-2) before the monocyte undergoes rTEM (Step-3). Blockade of JAM-C or increased vascular JAM-C expression induced higher levels of rTEM. (B) Blocking JAM-C 225.3 (white circles) had no effect on primary-TEM compared to conditions using an isotype control (grey circles). Blocking JAM-C 225.3 (white inverted triangles) led to increased rTEM of abluminal monocytes compared to isotype controls (grey inverted triangles). Data are presented as the mean of three fields ±SEM. Data shown is representative of three independent experiments.(N = 3). Decreased vascular JAM-C expression leads to a reduced occupation time of transmigrated monocytes. (C) Transmigrated monocytes on HUVECs with down-regulated JAM-C by siRNA transfection (JAM-C-neg) spent shorter intervals in the abluminal compartment (white triangles) compared to a sham transfection control (JAM-C-WT) (grey triangle). Data shown is representative of two independent experiments (N = 2). JAM-C blockade reduced the occupation interval of transmigrated monocyte in the abluminal compartment. (D) The time spent by individual monocyte in the abluminal compartment was assessed using 225.3 (white inverted triangles). This was shorter than co-cultures treated with an isotype control (grey inverted triangles). Data shown is representative of two independent experiments (N = 2). Increasing expression of JAM-C leads to a reduced occupation time of transmigrated monocytes. (E) HUVECS expressing JAM-C-1.8x (white triangles) had no effect on primary-TEM compared to EGFP-control transfected HUVECs (grey triangles). HUVECS expressing JAM-C-1.8x (white squares) led to increased rTEM of abluminal monocytes compared to the controls (grey squares). Data are presented as the mean of three fields ±SEM. Data shown is representative of three independent experiments (N = 3). Increasing expression of JAM-C also reduced the occupation interval of transmigrated monocyte in the abluminal compartment. (F) The time spent by individual monocytes in the abluminal compartment showed a reduction with increased expression of JAM-C-1.8x (white squares) and JAM-C-6.6x (white diamonds) compared to controls (grey squares). JAM-C-1.8x and JAM-C-6.6x had 1.8- and 6.6-times more expression than control JAM-C-WT respectively. Median values are marked. Mann–Whitney test was used for all dot plots statistical analyses. Data shown is representative of two independent experiments (N = 2).

Previous studies in humans have shown that disease onset and pathology are both marked by the accumulation of monocytic-derived cells [47]. We therefore decided to investigate the impact of JAM-C blockade on human monocyte adhesion and migration under flow conditions using a HUVEC co-culture system on a layer of collagen (Fig 4A). This system utilises hi-resolution phase contrast microscopy where leukocyte position on endothelial layers can be determined by phase appearance [48]. Captured monocytes on activated endothelial layers under flow typically have a phase-grey appearance as they migrate on luminal surfaces. However, monocytes that transmigrate switch to a phase-black appearance, allowing tracking of individual monocytes by two-dimensional imaging (S1 Movie).

We have previously shown that down-regulating JAM-C expression on HUVECs using siRNA or JAM-C blockade increased human monocyte rTEM. We conducted these studies using a cross-reactive anti-mouse JAM-C antibody [4]. The drawback of using this is that high concentrations of 50 μg/ml are required, as the affinity is relatively low against human JAM-C. We have subsequently developed a higher affinity, human specific anti-JAM-C antibody (225.3) determined by surface plasmon resonance technology (S1A Fig). Treatment of HUVECs with 10μg/ml of this new antibody was sufficient to induce rTEM of primary monocytes (Fig 4B). The level of rTEM reached 64% (±1.3%), nearly twice the level recorded with the isotype control antibody (32% ±6.6%). Similar to our previous findings [4], TEM was not affected with antibody 225.3 compared to isotype control (TEM at 60-mins was 84% ±1.3% and 91% ±1.5% respectively).

We then recorded the time individual monocytes spend in the abluminal compartment of HUVECs before rTEM. Monocytes typically occupied the abluminal compartment for 40-mins under control conditions, which was significantly reduced to 8-mins after JAM-C blockade with 225.3 antibody (P<0.005) (Fig 4C). HUVECs with knocked-down JAM-C expression by siRNA-1 transfection (JAM-C-neg) were also tested using this technique. JAM-C-neg HUVECs typically showed a 75%+ reduction from homeostatic levels in qPCR (S1B Fig) and flow cytometry studies (S1C Fig). Similar to results with antibody blockade, they also showed a reduction in monocyte abluminal occupation (30-mins) compared to the sham transfected control (48-mins, P<0.005), (Fig 4D). Together, these observations have confirmed that blocking JAM-C functionality through two independent mechanisms (blockade or reduced endogenous expression) leads to increased rTEM of monocytes.

During the onset of atherosclerosis, we found increased JAM-C expression at endothelial junctions, while endothelium in healthy vessels show low or marginal expression levels. Furthermore, previous studies have indicated that oxidised low-density protein (oxLDL); a known contributor to atherosclerosis, can lead to increased JAM-C expression on endothelial cells [11]. We therefore adapted a model that allowed us to investigate the impact of increasing JAM-C levels on monocyte rTEM in HUVECs [4]. For this, it was necessary to engineer a recombinant JAM-C-EGFP lentiviral construct (LV-JAM-C-EGFP) that allows JAM-C expression to be controlled in a dose-dependent manner [23] (S1 Appendix and S2 Fig). In order to confirm junctional integrity in HUVECs transfected with JAM-C-EGFP, we examined the distribution patterns of other junctional molecules including VE-Cadherin, PECAM-1, Occludin-1 and Claudin-5, as well as the PDZ domain proteins Afadin (AF) 6 and ZO-1 (S1D Fig). No differences were detected. It confirmed that JAM-C-EGFP transfection could increase the JAM-C load in HUVECs in a dose-dependent manner, without affecting the distribution of other junctional proteins.

To understand whether increased expression of JAM-C in HUVECs also had an effect on monocyte behaviour, we compared primary and rTEM using HUVECs transfected with JAM-C-EGFP (JAM-C-1.8x and JAM-C-6.6x) and a WT-control (JAM-C-WT). Rates of primary monocyte TEM under flow were comparable between JAM-C-WT and JAM-C-1.8x HUVECs, with levels of 98% ±1.4% and 95% ±1.4% respectively (Fig 4E). However, it was observed that increased JAM-C expression on HUVECs enhanced rTEM (Fig 4E). Surprisingly, we observed much higher levels of rTEM in JAM-C-1.8x HUVECs, when compared to the JAM-C-WT control group, which peaked at 87% (±1.7%) and 46% (±6.0%) respectively. This was also done at a higher concentration of JAM-C using JAM-C-6.6x HUVECs to determine if there is an additive effect to JAM-C-1.8x HUVECs. This significantly further reduced the time monocytes resided in the abluminal compartment (Fig 4F). Monocyte occupancy in the abluminal compartment on JAM-C-WT-GFP HUVECs was recorded at a median time of 36-mins, which was significantly reduced to 32-mins on JAM-C-1.8x HUVECs (P<0.05). Increasing the expression of JAM-C to 6.6x led to another significant reduction in the time of monocyte occupancy to 15-mins (P<0.01). Taken together, JAM-C participates in a one-way barrier for monocyte TEM, whereas the blockade or increased expression of JAM-C opens the gate leading to rTEM. Thus, JAM-C expression must be stringently controlled under homeostatic conditions.

### Monocyte Velocity is increased on HUVECs expressing high levels of JAM-C

Having established similar rTEM patterns with higher and lower levels of vascular JAM-C compared to homeostatic controls, we were keen to identify parameters that could differentiate between these effects. To this end, we measured the velocity of monocytes in the luminal and abluminal compartment on HUVECs with JAM-C expression ranging from close to zero expression (JAM-C-neg), to above physiological levels (JAM-C-6.6x).

**Fig 5:**
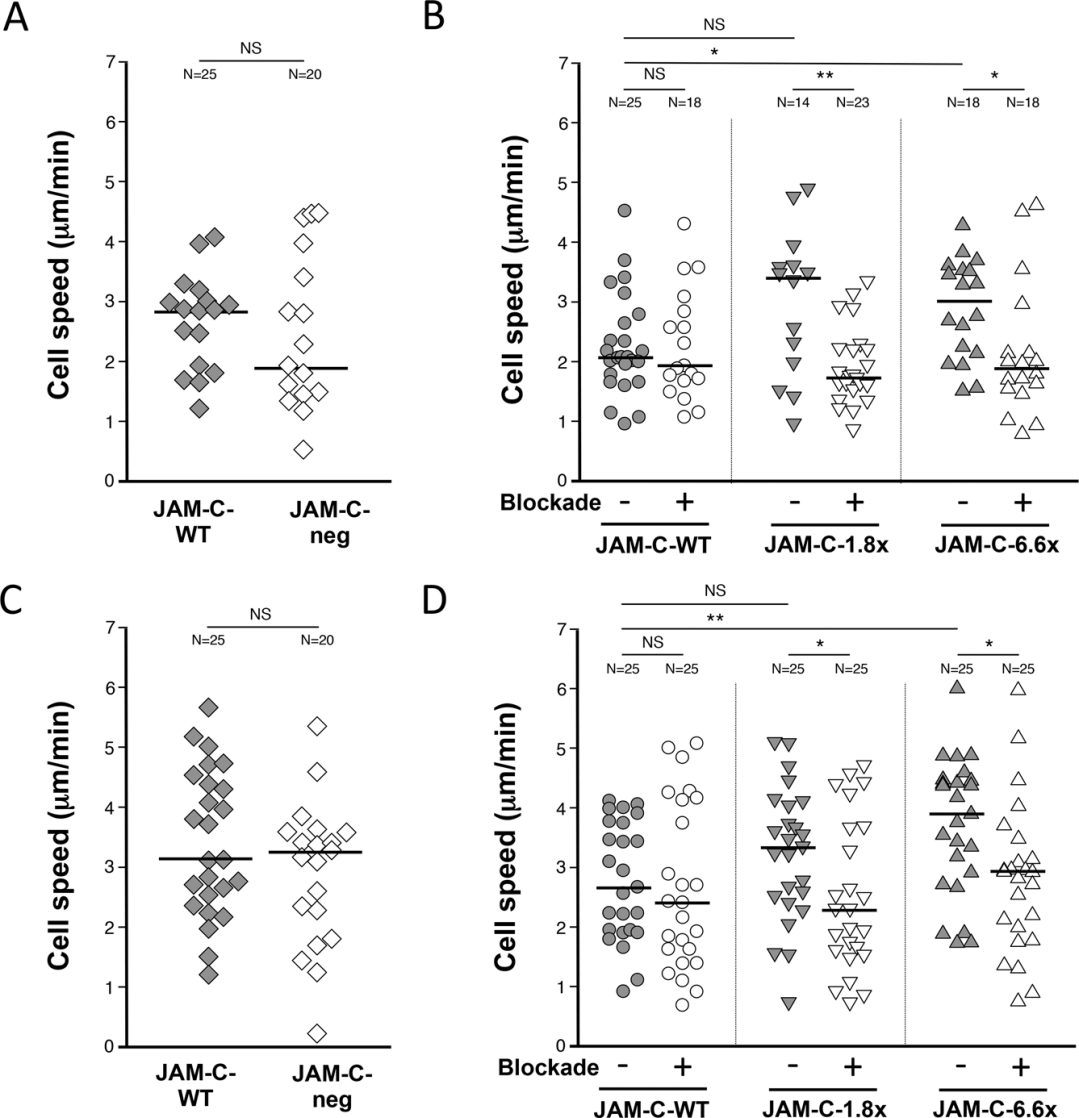
Monocytes migrate at higher velocities on HUVECs expressing increasing levels of JAM-C. The velocity of individual migrating monocytes was measured in the (A, B) abluminal and (C, D) abluminal compartment of HUVEC co-cultures under flow. (A, C) No significant effect was seen with monocytes on HUVECs with JAM-C-neg (white diamonds) compared to the sham transfected control JAM-C-WT HUVECs (grey diamonds) in both the (A) luminal and (C) abluminal compartment. Monocyte velocity was measured on JAM-C-WT (grey circles) JAM-C-1.8x (grey inverted triangles) and JAM-C-6.6x (grey triangles) HUVECs in the (B) luminal and (D) abluminal compartment. Blockade of JAM-C function using the 225.3 and isotype control were marked as ‘+’ and ‘-’ respectively. ‘N’ numbers indicate the number of monocytes analyzed per group. JAM-C-1.8x and JAM-C-6.6x had 1.8- and 6.6-times more expression than wild type levels (JAM-C-WT) respectively. Median values are marked. Mann–Whitney test was used for all dot plots statistical analyses (NS = not significant). Data shown is representative of two independent experiments (N = 2).

This was done by calculating the average velocity of individual monocytes on activated HUVEC monolayers immediately after a capture (luminal) or a TEM event (abluminal) (S3 Fig). Individual analysis for a number of monocytes in representative fields allowed the luminal and abluminal median velocity value to be calculated under different experimental conditions (S2 Movie). In the luminal compartment, no significant difference was observed in the velocity of monocytes on JAM-C-neg HUVECs versus JAM-C-WT expression levels (Fig 5A, 2.8 and 1.9 μm/min respectively, P>0.05). This technique was extended to include the anti-human JAM-C 225.3 antibody on JAM-C expressing HUVECs (Fig 5B). We have shown that JAM-C blockade had no effect on the velocity of monocyte migration on JAM-C-WT HUVECs compared to an isotype control (2.1 and 1.9 μm/min respectively, P>0.05). However, we identified a trend where HUVECs with enhanced JAM-C expression were able to support increasing velocities of monocyte migration. Notably, a significant increase was observed in JAM-C-6.6x HUVECs compared to WT controls (3.0 and 2.1 μm/min respectively, P<0.05). This effect was reversed using the antibody 225.3 in all HUVEC co-cultures with enhanced JAM-C expression. Monocyte velocities were reduced with 225.3 compared to the isotype control for both JAM-C-1.8x (3.4 and 1.7 μm/min respectively, P<0.01) and JAM-C-6.6x HUVEC co-culture models (3.0 and 1.9 μm/min respectively, P<0.05).

Almost identical observations were made for monocyte velocities in the abluminal compartment (Fig 5C and 5D). No significant difference was observed in the velocity of monocytes on HUVECs with JAM-C-neg HUVECs versus JAM-C-WT expression (Fig 5C, 3.1 and 3.3 μm/min respectively, P>0.05). Whilst no significant difference in monocyte velocity was seen with JAM-C blockade on JAM-C-WT compared to isotype control (Fig 5D, 2.4 and 2.7 μm/min respectively, P>0.05), significantly higher velocities were recorded on JAM-C-6.6x HUVECs compared to JAM-C-WT HUVECs (3.9 and 2.7 μm/min respectively, P<0.01). As with the luminal compartment, significant differences were recorded for monocyte velocity with JAM-C blockade versus their respective isotype control for both HUVEC co-culture models with JAM-C-1.8x (3.3 and 2.3 μm/min, P<0.05) and JAM-C-6.6x (3.9 and 3.0 μm/min, P<0.05).

In summary, our in-vitro model using single cell analysis has shown that HUVECs with increased JAM-C expression have the capacity to support monocyte migration at higher velocities. This increased monocyte velocity was reduced to control levels when treated with a functional blocking of JAM-C. However, these observations were distinct from those using normal and reduced levels of JAM-C where a further cumulative reduction in monocyte velocity did not occur.

### Accelerated regression of atherosclerotic plaques after JAM-C blockade is due to increased emigration of monocyte-derived cells

Having established an effect of JAM-C blockade on human monocyte rTEM by the in-vitro model, we decided to investigate the effect of blocking JAM-C in-vivo using our inflammatory, atherosclerosis regression model. Specifically, we asked whether the elevated levels of junctional JAM-C in the atherosclerotic plaques would actually promote or inhibit emigration of recruited monocytes, and if JAM-C blockade with a blocking antibody would enhance this process. Using this model, the dynamics of the regressing plaque could be attributed broadly to the reduced recruitment of circulating monocytes to the inflammatory lesion or increased egress of tissue-resident macrophages from the plaque into the circulation/lymphatics [41, 49].

**Fig 6:**
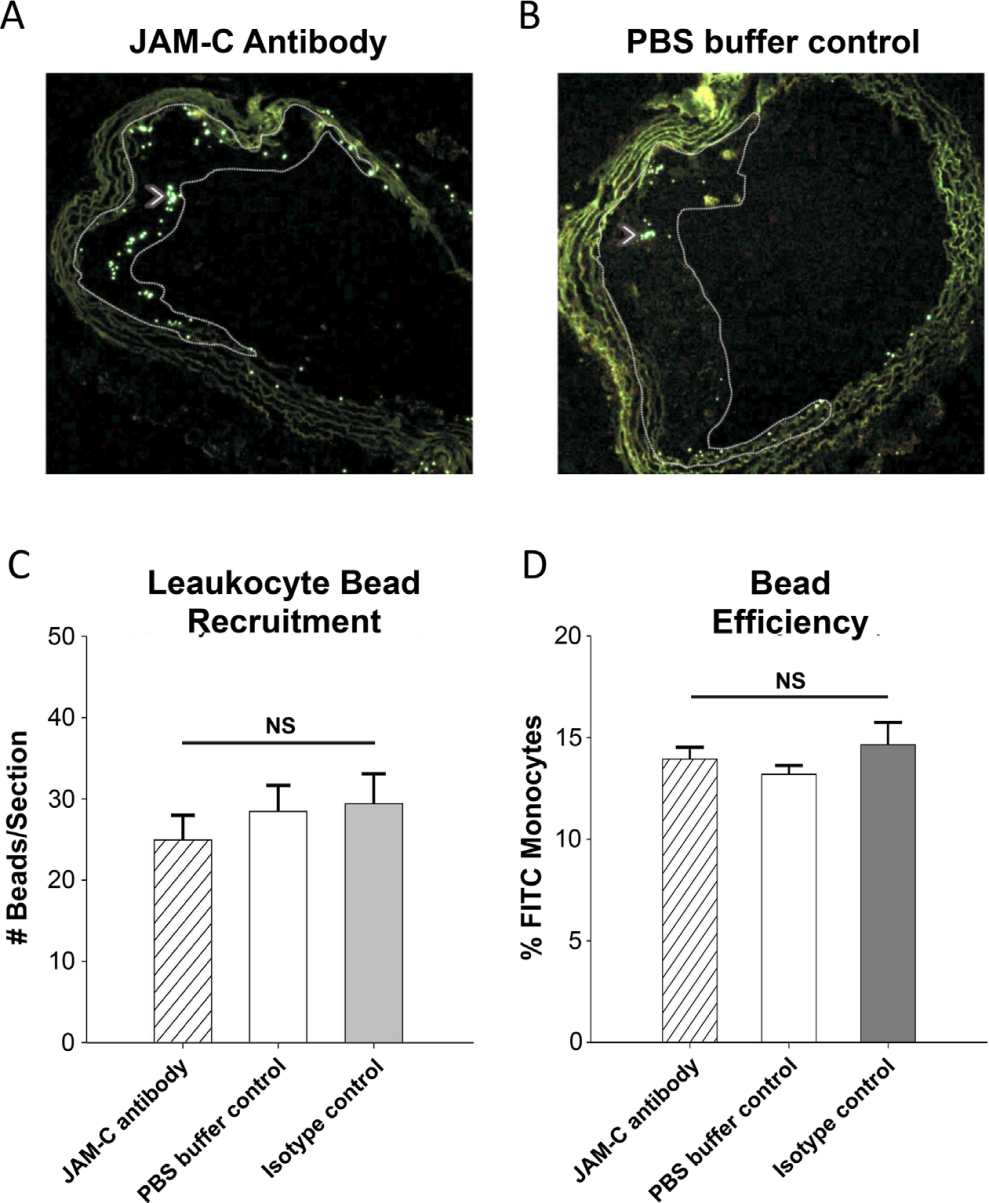
Monocyte bead recruitment assay. To assess monocyte recruitment, all recipients (anti-JAM-C, WT, or IgG control (not shown) were injected with fluorescent latex beads 48-hrs before sacrifice, which are taken up by circulating monocytes. Beads were identified by FITC+ fluorescence in the transplanted aortic arch sections, with representative sections shown for (A) anti-JAM-C antibody or (B) PBS buffer-control recipients. Beads are indicated with white arrowheads. (C) Beads were counted in aortic arch lesions and quantified by averaging #beads per arch section over at least 4 slides in the transplanted arch, similar to morphometrics. (D) Labeling efficiency of monocytes with the FITC beads was measured by blood draw 24-hrs after beads injection. Blood monocytes were analyzed for labeling efficiency by the percentage of FITC^+^ monocytes. Data are presented as the mean of three sections ±SEM (N = 6, NS = not significant).

To investigate these possibilities, we first utilized an in-vivo bead labeling technique to assess monocyte recruitment to plaque-bearing aortas after their transfer to a normolipidemic environment. Briefly, the WT mice that received the aortic transplant (recipient) were injected intravenously 2-days prior to sacrifice with fluorescent latex beads that then label circulating monocytes. These beads remain within the monocytes after entering tissues and therefore reflect their recruitment [39]. The number of labeled monocytes in sections of transplanted aortic arches was then quantified as a relative measurement of monocyte recruitment (Sample images in Fig 6A and 6B). No difference was observed between the recipient mice groups that were injected with anti-JAM-C antibody, PBS buffer-control control or an isotype control (Fig 6). This shows that JAM-C blockade did not change the recruitment of monocytes after plaques were exposed to normolipidemia (Fig 6C). To ensure equal labeling efficiency of circulating monocytes, blood was drawn from bead-injected mice 24-hrs after injection and analyzed by flow cytometry [36, 37, 39]. The percentage of fluorescent-labeled FITC+ cells was similar across all groups (Fig 6D, 11.5%-13.9%, P = 0.314,). Other leukocyte subpopulations served as negative labeling controls, and comprised less than 1% for neutrophils or CD45-negative cells (data not shown).

**Fig 7:**
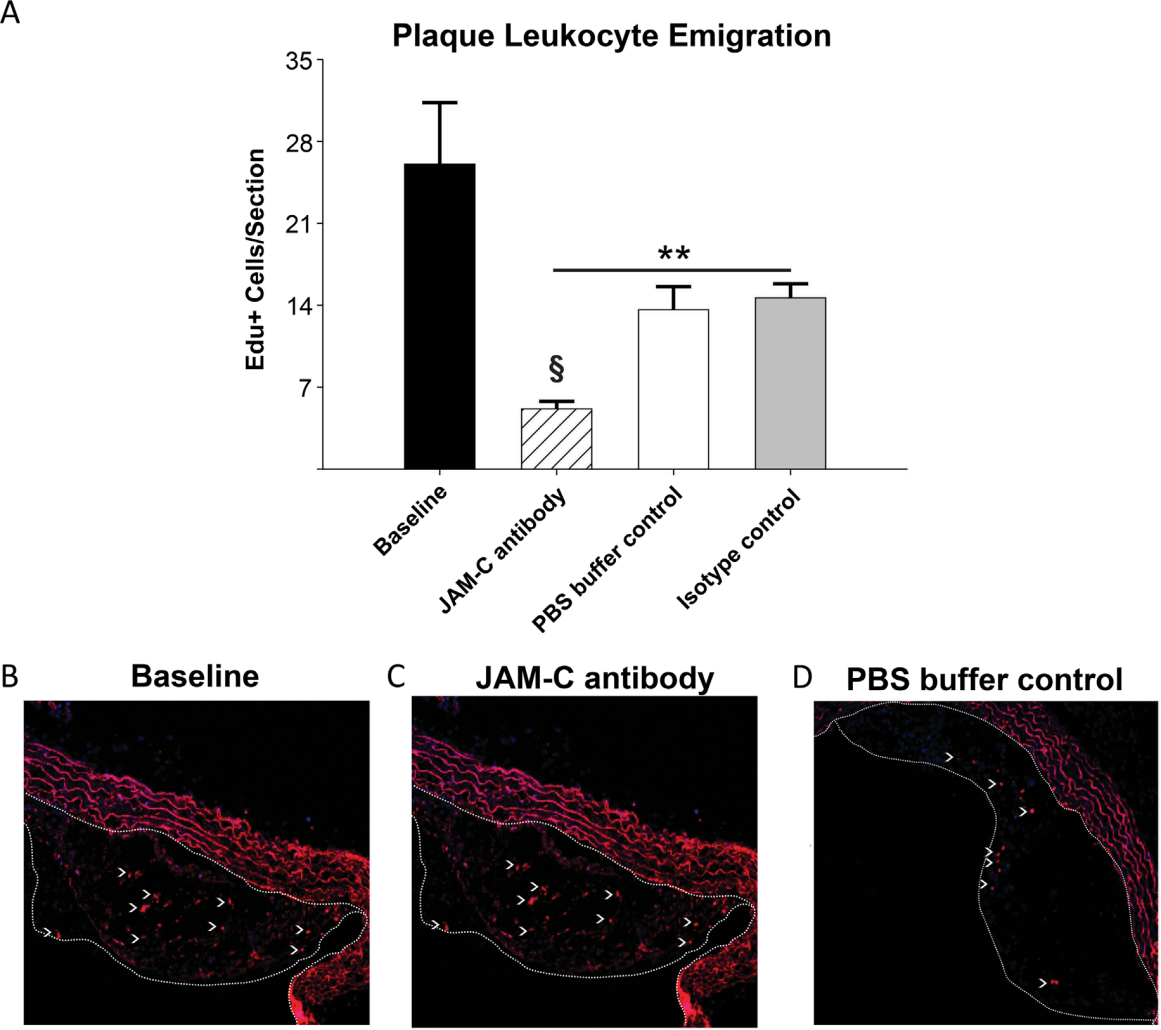
Increased atherosclerotic lesion regression following blockade of JAM-C is concurrent with increased leukocyte emigration from plaques. 48-hrs prior to transplantation or immediate sacrifice, all donor ApoE^-/-^ mice fed 12-wks of WD were injected with EdU (2mg/kg, i.p) to incorporate into DNA of monocyte precursors proliferating in the bone marrow. EdU-labeled circulating monocytes then enter the circulation and are recruited into atherosclerotic plaques. (A) Emigration from the plaques was quantified as the decrease in the number of EdU^+^ cells per section in the aortic arch plaques in baseline mice, compared to after transfer to the normolipidemic recipients. (B) Donor mice sacrificed at the transplant time point show robust EdU^+^ labeling in the aortic arch lesions. Recipient mice sacrificed 4-days after transplantation show significant reduction of EdU^+^ cells, indicating emigration during regression. (C) Recipient mice in which JAM-C was blocked via injections (JAM-C antibody) demonstrate even higher leukocyte egress than recipients treated with the isotype control (not shown) or (D) PBS buffer control. Lesions are outlined from endothelium with dashed white line. Data are presented as the mean value ±SEM (N = 6). ** = P<0.01 compared to baseline measurements. § = P<0.05 compared to WT and IgG recipients.

These results suggested that JAM-C blockade might accelerate atherosclerosis regression through an increase of the previously reported egress of monocytes/macrophages from plaques [36, 37]. To determine this experimentally, we employed a cell tracing technique using EdU, which is incorporated into the DNA of dividing cells [50, 51]. The ApoE^-/-^ donor mice were injected with EdU to label monocytes in the bone marrow prior to transplantation of the aorta. These labeled monocytes then exit into the circulation and enter tissues as EdU^+^ cells. Plaques in aortic arches of donor atherosclerotic mice showed a robust incorporation of EdU^+^ cells within the lesions. Baseline colonization of the aorta before transplantation was determined as 26.0 ±2.4 EdU^+^ cells/section (Fig 7A). In recipient, normolipidemic WT mice injected with anti-JAM-C antibody, the atherosclerotic plaques contained almost 3-times fewer EdU labeled cells compared to PBS buffer-control or isotype control antibody treated mice (Fig 7A, 7B, 7C and 7D. P<0.05). This experiment therefore excludes the possibility of contaminating CD68+ counts of smooth muscle cells [52, 53], as mentioned previously. As measured by staining for Ki-67, there was little evidence for cell proliferation in all recipient groups of aorta transplants during the short post-transplantation period, (data not shown). The change in the number of EdU+ cells in plaques therefore did not reflect different levels of local monocyte/macrophage proliferation in the lesions [54].

Overall, these results confirmed that in the normolipidemic environment where JAM-C junctional expression is increased in atherosclerotic aortas, emigration of monocyte-derived cells is enhanced by JAM-C blockade. This increased emigration upon JAM-C blockade correlates well with rTEM observed in-vitro with the primary human cells.

## Discussion

In the present study, we have explored the enigma of monocyte rTEM in-vitro and how this relates to the phenotypic outcome of JAM-C blockade in-vivo using atherosclerosis as a disease model. We have shown that high and low expression levels of JAM-C lead to increased rTEM of monocytes. This was done by using single cell analysis of human monocytes exposed to inflammatory endothelium under flow conditions. Consistent with the results from the low expression in-vitro studies, when aortic arches from hyperlipidemic ApoE^-/-^ mice were transplanted into anti-JAM-C treated normolipidemic animals; monocyte egression was increased, reducing the size of the atherosclerotic plaques.

Whilst we were initially surprised by the similar responses in-vitro to high and low levels of expression of JAM-C, we interpreted these data as revealing a precise homeostatic balance of JAM-C expression of monocyte retention in tissue by regulating unidirectional TEM [33, 55]. We studied the redistribution profile of JAM-C on the arteriosclerotic vessel wall using fluorescent microscopy in ApoE^-/-^ knockout mice. Within the atherosclerotic plaques of these mice, JAM-C is clearly observed in intercellular junctions of the endothelial vessel walls while it is almost absent in normal aortic tissue. This contrasts with venules, where JAM-C is expressed under resting conditions. Our study indicates that junctional expression of JAM-C in atherosclerotic arteries leads to monocyte retention as it approaches homeostatic levels found in venules [27]. Unexpectedly, we observed that increased JAM-C expression above homeostatic levels in HUVECs leads also to monocyte emigration. It is therefore conceivable that endothelium may upregulate JAM-C in an attempt to dampen the inflammatory response by actively returning tissue infiltrating inflammatory leukocytes back into the circulation.

**Fig 8:**
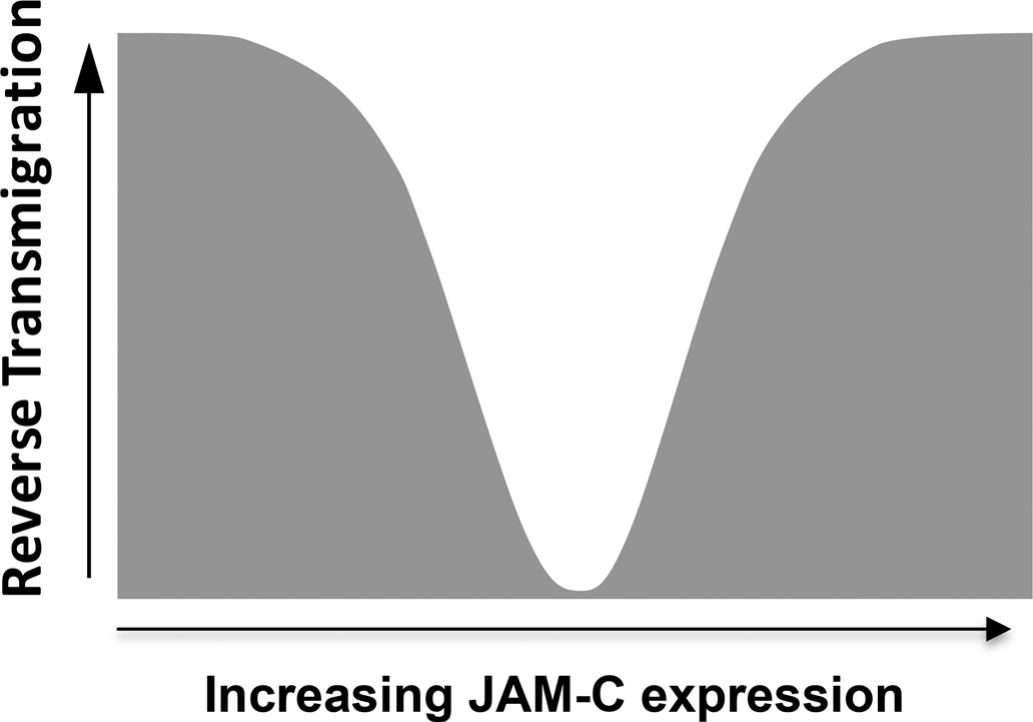
JAM-C expression level regulates monocyte retention and rTEM. As observed in both animal models and human disease, levels of vascular JAM-C can be decreased or increased during inflammation. JAM-C expression above and below homeostatic levels increased rTEM of monocytes.

An increase of JAM-C expression above homeostatic levels may alter the profile of other potential ligand interactions at the endothelial junction, thus affecting junctional integrity in-vivo. For example, a predominance of JAM-C/JAM-C homophilic interaction between two adjacent endothelial cells may prevail with increased expression levels, which are known to have a lower affinity and higher dynamics than JAM-C/JAM-B interactions [33]. This observation has led to an interesting scenario when considering the consequences of a shift in JAM-C expression (Fig 8). Whilst the total cellular load of JAM-C is important when considering other known ligand interactions (e.g. Macrophage-1 antigen (Mac-1), and Coxsackievirus and adenovirus receptor (CAR)) [26], JAM-C expression and the ratio of JAM-C:JAM-B within intercellular junctions may be the critical factor that dictate monocyte retention. Such a shift in JAM-C engagement could explain the increasing monocyte velocities on HUVECs with higher, but not lower, levels of JAM-C. A prevalence of ‘disengaged’ JAM-C has the potential to mediate endothelial JAM-C/monocyte Mac-1 interactions, thus regulating captured monocyte behaviour. However, the functional relevance of this remains unclear.

Based on our experimental observations in vitro, it is conceivable that endothelium may upregulate JAM-C in an attempt to dampen the inflammatory response by actively returning tissue infiltrating inflammatory leukocytes back into the circulation. However this has serious implications in pathologies associated with arteriole biology, where the opposite is true and can have catastrophic consequences. This raises the question about how JAM-C blockade or deficiency also induces monocyte rTEM. A potential role for JAM-C mediated platelet-monocyte crosstalk in rTEM was discounted, as adherent platelets are deposited on luminal surfaces immediately prior to TEM [4]. Furthermore, monocyte migration was also observed to increase with endothelial JAM-C expression in both luminal and abluminal compartments, implying this process is regulated bv vascular interactions. Interestingly, our previous studies have established that absence of JAM-C leaves JAM-B as an orphan molecule as it cannot form homophilic interactions between adjacent endothelial cells [4, 22, 27, 56]. This may give way to VE-Cadherin to exclusively control TEM, however rTEM remains unaffected [57, 58]. This mechanism seems to operate during a number of pathologies. For example, in ischemia/reperfusion injury, endothelial JAM-C on affected vessels mobilized away and shed from cell-cell contacts and was associated with increased neutrophil rTEM [27, 33, 56]. Consistent with these findings, we have confirmed that blockade and siRNA down-regulation of JAM-C on HUVEC monolayers in our co-culture system resulted in increased rates of rTEM. However, establishing a similar strategy for visualising or monitoring monocyte rTEM in-vivo has previously proven difficult, but has now been addressed by the use of an aortic transplantation atherosclerosis model.

As we have previously shown in this model, when plaques that develop in a hypercholesterolemic mouse are shifted to a WT, normolipidemic mouse, the content of the macrophage population significantly decreases [1] in association with emigration of the cells to both the systemic circulation and draining lymph nodes [36, 37]. It was also found that despite the reduction in hypercholesterolemia, there is continuing recruitment of monocytes [37], which, if unopposed by monocyte/macrophage emigration, will add to the inflammatory cell content of the lesions [38]. This suggests that if this worked in-vivo as it does in-vitro, its injection and blockade of function concurrent with plasma lipid reduction would improve atherosclerosis regression due to the enhancement of plaque monocytes and monocyte-derived cells rTEM, analogous to the in-vitro phenomena observed with monocytes migrating through endothelial cells. Indeed, this is what was found upon analysis of morphometrics and inflammatory macrophage content of aortic plaques. While they did not change in total plaque size compared to recipients, most likely because less extracellular matrix is destroyed by fewer and less inflammatory macrophages [44], anti-JAM-C treated mice specifically demonstrated reduced monocyte-derived cell content relative to other recipients through total and relative CD68^+^ inflammatory cell content.

Further strengthening the consistency between the in-vitro and in-vivo results are the leukocyte trafficking data in the mouse model. Monocyte recruitment, measured through a bead-labeling assay, showed no significant difference between the JAM-C, WT, or control IgG recipient groups in the number of circulating monocytes entering the plaques, similar to in-vitro data showing siRNA knockdown of JAM-C expression did not change primary monocyte TEM [4]. In this study, therefore, we have shown that there is a direct parallel between in-vitro primary-TEM of monocytes, and the in-vivo “recruitment” or primary-TEM of circulating monocytes into atherosclerotic lesions upon transplant to the normolipidemic environment.

We then raised the question of whether it was newly recruited monocytes that have undergone rTEM prior to differentiation to macrophages, or whether the emigrating cells included macrophages. A principal finding of the present study was the correlation between the in-vitro data demonstrating alterations in JAM-C expression and functional increases in monocyte rTEM (and migration velocity), and the in-vivo atherosclerosis model, where JAM-C blockade increased leukocyte emigration from plaques, which accelerated the regression of the inflammatory lesion.

Since this increased emigration resulted in reduced plaque macrophage content, it suggests that the blockade of JAM-C function increases monocyte and macrophage rTEM, preventing the accumulation of these cells in plaques. With regard to the effects on monocytes, this would also prevent their subsequent differentiation into macrophages. As for macrophage rTEM, this effect of JAM-C functional blockade is reminiscent of our recent studies on dendritic cells, another monocyte-derived cell, in which inhibiting JAM-C promoted dendritic cell rTEM and increased antigen presentation to T cells [19].

The role of JAM-C in leukocyte retention represents an extension in the functionality for the classical JAMs. A recent study has shown that endothelial deficiency in JAM-A, a close family member of JAM-C, did not affect vascular permeability, but profoundly affected leukocyte extravasation [59]. However, previous studies have shown that JAM-C plays a role in endothelial permeability [60, 61], indicating a capacity for different JAM family members to mediate similar yet opposing functionalities. Consistent with this hypothesis, we have shown that JAM-C not only plays a role in monocyte recruitment by regulating the velocity of luminal and abluminal migration, but it also plays an extended role in monocyte retention. The increasing velocity of monocytes with vascular JAM-C expression above homeostatic levels supports our model on junctional localisation of JAM-C. JAM-C that has moved out of junctions to other plasma membrane domains would therefore only be available above homeostatic levels, most likely induced by chronic inflammatory diseases. This implies that JAM-C can now interact with monocyte integrin ligands such as Mac-1 that would regulate the velocity of migration along the endothelial luminal and abluminal surfaces of endothelium. This has important implications when considering the role of JAM-C in the context of known inflammatory adhesion cascades. Vascular JAM-C interactions with captured leukocytes may be more prevalent on chronically inflamed endothelial surfaces, where leukocyte velocity correlates with disease chronicity, but can be reduced to basal homeostatic levels by JAM-C blockade.

Such observations must be considered when evaluating JAM-C as a therapeutic target, where an increased vascular JAM-C expression is perceived to have the same effect as blockade. However, our studies have shown other variables play a role, which requires further investigation. As discussed, increasing interactions with monocytes and vascular JAM-C may well be a pivotal factor that promotes disease progression. Furthermore, similar tactile interactions with increased JAM-C on smooth muscle cells and fibroblasts [13] may also participate in disrupting inflammatory resolution, indicating that expanded coculture or in-vivo models are required for future studies. One outcome from our study would be to profile the nature of JAM-C loading on inflamed tissues in different diseases. For example, JAM-C blockade on endothelium with very high levels of JAM-C may have little or no effect on net rTEM as both blockade and increased JAM-C supports increased rTEM.

This study has demonstrated the potential for JAM-C as a target for therapy in pathologies where accumulating leukocytes play a major role in disease progression. In atherosclerosis this may be particularly effective, given that lipid lowering treatment appears to stimulate some degree of rTEM of monocytes/macrophages [36, 37], so that further enhancement of this process would improve regression of disease and the resolution of plaque inflammation. Through the development of reagents specifically targeting molecules controlling leukocyte homing and accumulation, such as our JAM-C, successful therapy for patients suffering from chronic inflammatory diseases may be possible.

In summary, our findings have described a mechanistic role for JAM-C in regulating inflammation during normal physiology as well as pathology. Moreover, this study has contributed to a model where inflammatory diseases may be profiled for chronicity based on vascular and tissue JAM-C expression.

## Supporting Information

S1 Appendix Recombinant JAM-C is stably expressed and localizes to junctions of HUVEC monolayers. (DOCX)

S1 Fig Functional Blockade of JAM-C and phenotypic effect on cultured HUVECs. (A) Characterization of the novel anti-human JAM-C antibody 225.3. Biacore evaluation of anti-JAM-C Abs on immobilized soluble JAM-C comparing the anti-mouse JAM-C cross-reactive H36 Abs with the anti-human JAM-C 225.3. (B) Validation of JAM-C down-regulation on HUVECs after siRNA delivery by qPCR. Inhibition of JAM-C expression on cultured HUVECs after transfection with huJAM-C siRNA 1 and 2, when compared to control siRNA and mock control. All values were normalized to the expression levels of human beta-actin, beta-tubulin, and GAPDH. JAM-C siRNA 1 silenced > 75% of the huJAM-C mRNA in HUVECs after transfection and was used for all monocyte coculture experiments. Bars represent mean ± standard deviation (SD) (3 experiments, each condition in triplicate). P values were calculated compared to control siRNA (* = P<0.05; ** = P<0.01). (C) Validation of JAM-C down-regulation on HUVECs by flow cytometry. JAM-C expression on HUVECs was reduced after transfection with siRNA1 (pink) and 2 (blue), compared to JAM-C expression on non-transfected HUVECs (green). Expression of JAM-C on HUVECs transfected with control siRNA (blue) and non-transfected HUVECs remained comparable. An isotype control was included in all experiments (black). Histograms are representative of at least 2 experiments. (D) Overexpression of recombinant JAM-C does not affect distribution of other junctional proteins. Localization of the junctional proteins VE-Cadherin. PECAM-1. Occludin-1, Claudin-5, AF6 and ZO-1 were examined using confocal microscopy. No differences were observed between cells transfected with the control EGFP (marked as ‘-‘) and JAM-C-EGFP constructs (marked as ‘+‘). Images are representative of at least 4 independent experiments (N = 4). All antibody isotype controls included for immunofluorescence showed no staining (data not shown).
(TIF)

S2 Fig Functional expression of JAM-C-EGFP on cultured HUVECs after stimulation. (A) Cultured HUVEC monolayers were stimulated with TNF-alpha and fixed at set time-points of 0 (unstimulated), 1- and 4-hrs. Human HUVECs were stained for human JAM-C using antibody 225.3 or an isotype control. JAM-C distribution remained unchanged throughout the 4-hr time-course with JAM-C remaining mostly in the junctions. The isotype control antibody showed no staining (data not shown). (B) Cultured HUVEC monolayers were transfected with JAM-C-EGFP lentivirus and stimulated with TNF-alpha at 0 (unstimulated), 1- and 4-hrs. Distribution of JAM-C-EGFP was similar to endogenous JAM-C and localized to intercellular junctions but also showed accumulation intracellularly. (C) Analysis by flow cytometry established total JAM-C expression in 73–76% of HUVECs transfected with JAM-C-EGFP and this increased in a linear fashion when compared to total JAM-C. Identical profiles were seen at 0-, 1- and 4-hrs after stimulation with TNF-alpha. (D) Increased total JAM-C expression using the JAM-C-EGFP construct (squares) was typically ~2-times higher than normal endogenous JAM-C expression (circles). (E) Comparison of JAM-C expression to the starting level of expression (MFI) in the endogenous (circles), total (squares) and JAM-C-EGFP populations (triangles) confirmed expression levels in each population were stable and remained unchanged up to 24-hrs. (F) An example set of flow cytometry profiles illustrating how total JAM-C expression increases with LV-JAM-C-EGFP load (G) Titration of LV-JAM-C-EGFP on cultured HUVECs. Flow cytometry studies indicated lentivirus JAM-C-EGFP preparation on cultured HUVECs stimulated with TNF-alpha increased total surface JAM-C expression in a dose-dependent manner. Titrations tested in this experiment were 1:100 (white circles), 1:500 (grey circles), 1:1000 (white triangle), 1:2000 (grey triangle), 1:5000 (white square) and a no virus control (grey square). Profiles of JAM-C expression remained constant at all concentrations up to 24-hrs for each concentration. (H) The MFI of total JAM-C expression increased in a linear fashion with LV load. Dilution rates of 1/1000 and 1/100 were used to generate HUVECs with 1.8 (JAM-C-1.8x) and 6.6 fold increase (JAM-C-6.6x) above homeostatic JAM-C levels. (I) Increasing JAM-C expression had no effect on VE-cadherin expression. While JAM-C expression was increased on HUVECs, VE-cadherin remained unaffected. (J) VE-cadherin remained similarly unaffected on HUVECs after 4-hrs stimulation with TNF-alpha (conditions used in flow assay). Data shown is representative of two independent experiments (N = 2).
(TIF)

S3 Fig Single cell-tracking of individual monocytes on activated HUVECs under flow. (A, B) Images are for two example monocytes (A and B) and contain representative cell tracking paths, plus a corresponding summary table detailing cell position and velocity analysis. The Capture point from flow, and cell position is represented by circles denoting the time (mins) and XY position. Circles with a black or red border denote a monocyte in the luminal and abluminal compartment respectively. The track direction is represented by green and red tracks for monocyte-A and -B respectively. An early timepoint of 15-mins was selected for the images in order to illustrate the tracking paths associated with each monocyte in different compartments. The extended track of Monocyte-A and–B can be observed as Monocyte-2 and -3 respectively in S2 Movie. The XY pixel coordinates and time for each individual monocyte were summarised in tables, allowing luminal and abluminal velocities to be recorded (data sets marked in black and red respectively). The average velocity for a given monocyte in each compartment was calculated over a 3-min period (highlighted in yellow). This was done with values immediately after monocyte capture (luminal velocity) or TEM (abluminal velocity).
(TIF)

S1 Movie TEM and rTEM of an adherent monocyte on activated HUVECs under flow. An adherent monocyte undergoing capture, firm adhesion and migration on luminal surfaces has a phase-grey appearance using phase contrast micrscopy (highlighted with intermittent red). As the monocyte transmigrates across the endothelial cell junction, there is change in phase appearance from grey to black (highlighted with intermittent green). Transmigrated monocytes remain phase-black for the duration of their occupancy until they undergo rTEM back onto luminal surfaces and revert to a phase-grey appearance.
(MP4)

S2 Movie Individual cell tracking analysis (position and velocity) of multiple monocytes within a single field. Cell position and velocity were recorded for selected individual monocyte captured from flow onto activated HUVEC luminal surfaces. This was typically done for 25 monocytes in an individual field where monocyte position and XY coordinates were recorded at 1-min intervals. Circles with a black or red border denote a monocyte in the luminal and abluminal compartment respectively. Each colored track corresponds to an individual monocyte and a data summary table describing event, XY position and velocity, as described in S3 Fig.
(MP4)

## References

[1] Moore KJ, Sheedy FJ, Fisher EA. Macrophages in atherosclerosis: a dynamic balance. Nat Rev Immunol. 2013;13(10):709–21. 10.1038/nri3520 23995626 PMC4357520

[2] Merched AJ, Ko K, Gotlinger KH, Serhan CN, Chan L. Atherosclerosis: evidence for impairment of resolution of vascular inflammation governed by specific lipid mediators. FASEB J. 2008;22(10):3595–606. 10.1096/fj.08-112201 18559988 PMC2537438

[3] Yla-Herttuala S, Bentzon JF, Daemen M, Falk E, Garcia-Garcia HM, Herrmann J, et al Stabilization of atherosclerotic plaques: an update. Eur Heart J. 2013;34(42):3251–8. 10.1093/eurheartj/eht301 23966311

[4] Bradfield PF, Scheiermann C, Nourshargh S, Ody C, Luscinskas FW, Rainger GE, et al JAM-C regulates unidirectional monocyte transendothelial migration in inflammation. Blood. 2007;110(7):2545–55. 17625065 10.1182/blood-2007-03-078733 PMC1988941

[5] Nourshargh S, Alon R. Leukocyte migration into inflamed tissues. Immunity. 2014;41(5):694–707. 10.1016/j.immuni.2014.10.008 25517612

[6] Garrido-Urbani S, Bradfield PF, Imhof BA. Tight junction dynamics: the role of junctional adhesion molecules (JAMs). Cell Tissue Res. 2014;355(3):701–15. 10.1007/s00441-014-1820-1 24595739

[7] Bazzoni G. The JAM family of junctional adhesion molecules. Curr Opin Cell Biol. 2003;15(5):525–30. 14519386 10.1016/s0955-0674(03)00104-2

[8] Aurrand-Lions M, Duncan L, Ballestrem C, Imhof BA. JAM-2, a novel immunoglobulin superfamily molecule, expressed by endothelial and lymphatic cells. J Biol Chem. 2001;276(4):2733–41. 11053409 10.1074/jbc.M005458200

[9] Ebnet K, Suzuki A, Ohno S, Vestweber D. Junctional adhesion molecules (JAMs): more molecules with dual functions? J Cell Sci. 2004;117(Pt 1):19–29. 14657270 10.1242/jcs.00930

[10] Weber C, Fraemohs L, Dejana E. The role of junctional adhesion molecules in vascular inflammation. Nat Rev Immunol. 2007;7(6):467–77. 17525755 10.1038/nri2096

[11] Keiper T, Al-Fakhri N, Chavakis E, Athanasopoulos AN, Isermann B, Herzog S, et al The role of junctional adhesion molecule-C (JAM-C) in oxidized LDL-mediated leukocyte recruitment. FASEB J. 2005;19(14):2078–80. 16195363 10.1096/fj.05-4196fje

[12] Palmer G, Busso N, Aurrand-Lions M, Talabot-Ayer D, Chobaz-Peclat V, Zimmerli C, et al Expression and function of junctional adhesion molecule-C in human and experimental arthritis. Arthritis Res Ther. 2007;9(4):R65 17612407 10.1186/ar2223 PMC2206366

[13] Rabquer BJ, Pakozdi A, Michel JE, Gujar BS, Haines GK 3rd, Imhof BA, et al Junctional adhesion molecule C mediates leukocyte adhesion to rheumatoid arthritis synovium. Arthritis Rheum. 2008;58(10):3020–9. 10.1002/art.23867 18821692 PMC2911024

[14] Vonlaufen A, Aurrand-Lions M, Pastor CM, Lamagna C, Hadengue A, Imhof BA, et al The role of junctional adhesion molecule C (JAM-C) in acute pancreatitis. J Pathol. 2006;209(4):540–8. 16767690 10.1002/path.2007

[15] Zimmerli C, Lee BP, Palmer G, Gabay C, Adams R, Aurrand-Lions M, et al Adaptive immune response in JAM-C-deficient mice: normal initiation but reduced IgG memory. J Immunol. 2009;182(8):4728–36. 10.4049/jimmunol.0803892 19342649

[16] Chavakis T, Keiper T, Matz-Westphal R, Hersemeyer K, Sachs UJ, Nawroth PP, et al The junctional adhesion molecule-C promotes neutrophil transendothelial migration in vitro and in vivo. J Biol Chem. 2004;279(53):55602–8. 15485832 10.1074/jbc.M404676200

[17] Shagdarsuren E, Djalali-Talab Y, Aurrand-Lions M, Bidzhekov K, Liehn EA, Imhof BA, et al Importance of junctional adhesion molecule-C for neointimal hyperplasia and monocyte recruitment in atherosclerosis-prone mice-brief report. Arterioscler Thromb Vasc Biol. 2009;29(8):1161–3. 10.1161/ATVBAHA.109.187898 19520977

[18] Donate C, Ody C, McKee T, Ruault-Jungblut S, Fischer N, Ropraz P, et al Homing of human B cells to lymphoid organs and B-cell lymphoma engraftment are controlled by cell adhesion molecule JAM-C. Cancer Res. 2013;73(2):640–51. 10.1158/0008-5472.CAN-12-1756 23221386

[19] Ballet R, Emre Y, Jemelin S, Charmoy M, Tacchini-Cottier F, Imhof BA. Blocking junctional adhesion molecule C enhances dendritic cell migration and boosts the immune responses against Leishmania major. PLoS Pathog. 2014;10(12):e1004550 10.1371/journal.ppat.1004550 25474593 PMC4256467

[20] Langer HF, Orlova VV, Xie C, Kaul S, Schneider D, Lonsdorf AS, et al A novel function of junctional adhesion molecule-C in mediating melanoma cell metastasis. Cancer Res. 2011;71(12):4096–105. 10.1158/0008-5472.CAN-10-2794 21593193 PMC3117056

[21] Ghislin S, Obino D, Middendorp S, Boggetto N, Alcaide-Loridan C, Deshayes F. Junctional adhesion molecules are required for melanoma cell lines transendothelial migration in vitro. Pigment Cell Melanoma Res. 2011;24(3):504–11. 10.1111/j.1755-148X.2011.00856.x 21466663

[22] Woodfin A, Voisin MB, Beyrau M, Colom B, Caille D, Diapouli FM, et al The junctional adhesion molecule JAM-C regulates polarized transendothelial migration of neutrophils in vivo. Nat Immunol. 2011;12(8):761–9. 10.1038/ni.2062 21706006 PMC3145149

[23] Sircar M, Bradfield PF, Aurrand-Lions M, Fish RJ, Alcaide P, Yang L, et al Neutrophil transmigration under shear flow conditions in vitro is junctional adhesion molecule-C independent. J Immunol. 2007;178(9):5879–87. 17442972 10.4049/jimmunol.178.9.5879

[24] Huttenlocher A, Poznansky MC. Reverse leukocyte migration can be attractive or repulsive. Trends Cell Biol. 2008;18(6):298–306. 10.1016/j.tcb.2008.04.001 18468440 PMC2435406

[25] Buckley CD, Ross EA, McGettrick HM, Osborne CE, Haworth O, Schmutz C, et al Identification of a phenotypically and functionally distinct population of long-lived neutrophils in a model of reverse endothelial migration. J Leukoc Biol. 2006;79(2):303–11. 16330528 10.1189/jlb.0905496

[26] Bradfield PF, Nourshargh S, Aurrand-Lions M, Imhof BA. JAM family and related proteins in leukocyte migration (Vestweber series). Arterioscler Thromb Vasc Biol. 2007;27(10):2104–12. 17615384 10.1161/ATVBAHA.107.147694

[27] Colom B, Bodkin JV, Beyrau M, Woodfin A, Ody C, Rourke C, et al Leukotriene B-Neutrophil Elastase Axis Drives Neutrophil Reverse Transendothelial Cell Migration In Vivo. Immunity. 2015. 10.1016/j.immuni.2015.05.010 PMC4504024 26047922

[28] Wall RT, Harker LA, Quadracci LJ, Striker GE. Factors influencing endothelial cell proliferation in vitro. J Cell Physiol. 1978;96(2):203–13. 670305 10.1002/jcp.1040960209

[29] Gimbrone MA Jr., Cotran RS, Folkman J. Endothelial regeneration: studies with human endothelial cells in culture. Series haematologica (1968). 1973;6(4):453–5. 4794930

[30] Jaffe EA, Nachman RL, Becker CG, Minick CR. Culture of human endothelial cells derived from umbilical veins. Identification by morphologic and immunologic criteria. The Journal of clinical investigation. 1973;52(11):2745–56. 4355998 10.1172/JCI107470 PMC302542

[31] Nachman RL, Jaffe EA. Endothelial cell culture: beginnings of modern vascular biology. The Journal of clinical investigation. 2004;114(8):1037–40. 15489946 10.1172/JCI23284 PMC522268

[32] Vandesompele J, De Preter K, Pattyn F, Poppe B, Van Roy N, De Paepe A, et al Accurate normalization of real-time quantitative RT-PCR data by geometric averaging of multiple internal control genes. Genome Biol. 2002;3(7):Research0034 12184808 10.1186/gb-2002-3-7-research0034 PMC126239

[33] Lamagna C, Meda P, Mandicourt G, Brown J, Gilbert RJ, Jones EY, et al Dual interaction of JAM-C with JAM-B and alpha(M)beta2 integrin: function in junctional complexes and leukocyte adhesion. Mol Biol Cell. 2005;16(10):4992–5003. 16093349 10.1091/mbc.E05-04-0310 PMC1237098

[34] Shaw SK, Bamba PS, Perkins BN, Luscinskas FW. Real-time imaging of vascular endothelial-cadherin during leukocyte transmigration across endothelium. J Immunol. 2001;167(4):2323–30. 11490021 10.4049/jimmunol.167.4.2323

[35] Chereshnev I, Trogan E, Omerhodzic S, Itskovich V, Aguinaldo JG, Fayad ZA, et al Mouse model of heterotopic aortic arch transplantation. J Surg Res. 2003;111(2):171–6. 12850459 10.1016/s0022-4804(03)00039-8

[36] Trogan E, Feig JE, Dogan S, Rothblat GH, Angeli V, Tacke F, et al Gene expression changes in foam cells and the role of chemokine receptor CCR7 during atherosclerosis regression in ApoE-deficient mice. Proc Natl Acad Sci U S A. 2006;103(10):3781–6. 16537455 10.1073/pnas.0511043103 PMC1450154

[37] Llodra J, Angeli V, Liu J, Trogan E, Fisher EA, Randolph GJ. Emigration of monocyte-derived cells from atherosclerotic lesions characterizes regressive, but not progressive, plaques. Proc Natl Acad Sci U S A. 2004;101(32):11779–84. 15280540 10.1073/pnas.0403259101 PMC511052

[38] Potteaux S, Gautier EL, Hutchison SB, van Rooijen N, Rader DJ, Thomas MJ, et al Suppressed monocyte recruitment drives macrophage removal from atherosclerotic plaques of Apoe-/- mice during disease regression. The Journal of clinical investigation. 2011;121(5):2025–36. 10.1172/JCI43802 21505265 PMC3083793

[39] Tacke F, Alvarez D, Kaplan TJ, Jakubzick C, Spanbroek R, Llodra J, et al Monocyte subsets differentially employ CCR2, CCR5, and CX3CR1 to accumulate within atherosclerotic plaques. The Journal of clinical investigation. 2007;117(1):185–94. 17200718 10.1172/JCI28549 PMC1716202

[40] Feig JE, Pineda-Torra I, Sanson M, Bradley MN, Vengrenyuk Y, Bogunovic D, et al LXR promotes the maximal egress of monocyte-derived cells from mouse aortic plaques during atherosclerosis regression. The Journal of clinical investigation. 2010;120(12):4415–24. 10.1172/JCI38911 21041949 PMC2993578

[41] Distel E, Barrett TJ, Chung K, Girgis NM, Parathath S, Essau CC, et al miR33 inhibition overcomes deleterious effects of diabetes mellitus on atherosclerosis plaque regression in mice. Circ Res. 2014;115(9):759–69. 10.1161/CIRCRESAHA.115.304164 25201910 PMC4194153

[42] Bartolini B, Thelin MA, Svensson L, Ghiselli G, van Kuppevelt TH, Malmstrom A, et al Iduronic acid in chondroitin/dermatan sulfate affects directional migration of aortic smooth muscle cells. PLoS One. 2013;8(7):e66704 10.1371/journal.pone.0066704 23843960 PMC3699603

[43] Liu SQ, Goldman J. Role of blood shear stress in the regulation of vascular smooth muscle cell migration. IEEE Trans Biomed Eng. 2001;48(4):474–83. 11322535 10.1109/10.915714

[44] Parathath S, Grauer L, Huang LS, Sanson M, Distel E, Goldberg IJ, et al Diabetes adversely affects macrophages during atherosclerotic plaque regression in mice. Diabetes. 2011;60(6):1759–69. 10.2337/db10-0778 21562077 PMC3114401

[45] Murphy AJ, Akhtari M, Tolani S, Pagler T, Bijl N, Kuo CL, et al ApoE regulates hematopoietic stem cell proliferation, monocytosis, and monocyte accumulation in atherosclerotic lesions in mice. The Journal of clinical investigation. 2011;121(10):4138–49. 10.1172/JCI57559 21968112 PMC3195472

[46] Swirski FK, Libby P, Aikawa E, Alcaide P, Luscinskas FW, Weissleder R, et al Ly-6Chi monocytes dominate hypercholesterolemia-associated monocytosis and give rise to macrophages in atheromata. The Journal of clinical investigation. 2007;117(1):195–205. 17200719 10.1172/JCI29950 PMC1716211

[47] Ross R. Atherosclerosis—an inflammatory disease. N Engl J Med. 1999;340(2):115–26. 9887164 10.1056/NEJM199901143400207

[48] Luu NT, Rainger GE, Nash GB. Kinetics of the different steps during neutrophil migration through cultured endothelial monolayers treated with tumour necrosis factor-alpha. J Vasc Res. 1999;36(6):477–85. 10629423 10.1159/000025690

[49] van Gils JM, Ramkhelawon B, Fernandes L, Stewart MC, Guo L, Seibert T, et al Endothelial expression of guidance cues in vessel wall homeostasis dysregulation under proatherosclerotic conditions. Arterioscler Thromb Vasc Biol. 2013;33(5):911–9. 10.1161/ATVBAHA.112.301155 23430612 PMC3647028

[50] Nagareddy PR, Kraakman M, Masters SL, Stirzaker RA, Gorman DJ, Grant RW, et al Adipose tissue macrophages promote myelopoiesis and monocytosis in obesity. Cell Metab. 2014;19(5):821–35. 10.1016/j.cmet.2014.03.029 24807222 PMC4048939

[51] Zhu SN, Chen M, Jongstra-Bilen J, Cybulsky MI. GM-CSF regulates intimal cell proliferation in nascent atherosclerotic lesions. J Exp Med. 2009;206(10):2141–9. 10.1084/jem.20090866 19752185 PMC2757868

[52] Rong JX, Shapiro M, Trogan E, Fisher EA. Transdifferentiation of mouse aortic smooth muscle cells to a macrophage-like state after cholesterol loading. Proc Natl Acad Sci U S A. 2003;100(23):13531–6. 14581613 10.1073/pnas.1735526100 PMC263848

[53] Shankman LS, Gomez D, Cherepanova OA, Salmon M, Alencar GF, Haskins RM, et al KLF4-dependent phenotypic modulation of smooth muscle cells has a key role in atherosclerotic plaque pathogenesis. Nat Med. 2015;21(6):628–37. 10.1038/nm.3866 25985364 PMC4552085

[54] Robbins CS, Hilgendorf I, Weber GF, Theurl I, Iwamoto Y, Figueiredo JL, et al Local proliferation dominates lesional macrophage accumulation in atherosclerosis. Nat Med. 2013;19(9):1166–72. 10.1038/nm.3258 23933982 PMC3769444

[55] Springer TA. Traffic signals for lymphocyte recirculation and leukocyte emigration: the multistep paradigm. Cell. 1994;76(2):301–14. 7507411 10.1016/0092-8674(94)90337-9

[56] Scheiermann C, Colom B, Meda P, Patel NS, Voisin MB, Marrelli A, et al Junctional adhesion molecule-C mediates leukocyte infiltration in response to ischemia reperfusion injury. Arterioscler Thromb Vasc Biol. 2009;29(10):1509–15. 10.1161/ATVBAHA.109.187559 19574560 PMC2746810

[57] Wessel F, Winderlich M, Holm M, Frye M, Rivera-Galdos R, Vockel M, et al Leukocyte extravasation and vascular permeability are each controlled in vivo by different tyrosine residues of VE-cadherin. Nat Immunol. 2014;15(3):223–30. 10.1038/ni.2824 24487320

[58] Sidibe A, Imhof BA. VE-cadherin phosphorylation decides: vascular permeability or diapedesis. Nat Immunol. 2014;15(3):215–7. 10.1038/ni.2825 24549064

[59] Schmitt MM, Megens RT, Zernecke A, Bidzhekov K, van den Akker NM, Rademakers T, et al Endothelial junctional adhesion molecule-a guides monocytes into flow-dependent predilection sites of atherosclerosis. Circulation. 2014;129(1):66–76. 10.1161/CIRCULATIONAHA.113.004149 24065611

[60] Li X, Stankovic M, Lee BP, Aurrand-Lions M, Hahn CN, Lu Y, et al JAM-C induces endothelial cell permeability through its association and regulation of {beta}3 integrins. Arterioscler Thromb Vasc Biol. 2009;29(8):1200–6. 10.1161/ATVBAHA.109.189217 19461049

[61] Orlova VV, Economopoulou M, Lupu F, Santoso S, Chavakis T. Junctional adhesion molecule-C regulates vascular endothelial permeability by modulating VE-cadherin-mediated cell-cell contacts. The Journal of experimental medicine. 2006;203(12):2703–14. 17116731 10.1084/jem.20051730 PMC2118160

