# Supplementary material for: Divergent JAM-C Expression Accelerates Monocyte-Derived Cell Exit from Atherosclerotic Plaques
Source: PLoS One. 2016 Jul 21;11(7):e0159679. doi: 10.1371/journal.pone.0159679 (PMC4956249; doi:10.1371/journal.pone.0159679)
Supplement: S1 Appendix — (DOCX) [file pone.0159679.s001.docx]

**S1 Appendix**

**Recombinant JAM-C is stably expressed and localizes to junctions of HUVEC monolayers.**

In order to determine the profiles of JAM-C/JAM-C-EGFP on HUVECs, both molecules were measured during the course of TNFα stimulation. To generate JAM-C-EGFP, EGFP was inserted into the hinge region of JAM-C between the membrane proximal C2 domain and the trans-membrane region to avoid obstruction of the polarizing, cytoplasmic PDZ domain [1, 2]. Microscopy revealed that recombinant JAM-C-EGFP localized to cell-cell contacts similar to endogenous JAM-C. We found that this JAM-C-EGFP behaves similar to endogenous JAM-C (S2 Fig) More importantly, upon 4-hr TNFα activation (timescale used for stimulation), neither the distribution nor the expression level of recombinant JAM-C-EGFP changed compared to endogenous JAM-C (S2A,B Fig). However, there was a clear indication that a proportion of JAM-C-EGFP remained internalised and not on cellular surfaces, indicating the critical need of measuring total JAM-C surface expression in these experiments. We found that endogenous and transfected levels remained constant for the duration of TNFα stimulation (S2C Fig). This indicates that JAM-C-EGFP also behaves in a similar fashion to endogenous JAM-C. LV-JAM-C-EGFP transfection at the baseline level resulted in a 1.8-fold (JAM-C-1.8x) increase in JAM-C expression and remained stable over 24-hrs after TNFα activation (S2D,E Fig).

Using this model, we were able to extend the range of LV-JAM-C-EGFP dilutions to establish the feasibility limits of JAM-C transfection by looking at the increase in JAM-C expression with JAM-C-EGFP transfection (S2F Fig). This remained unchanged throughout the course of TNFα stimulation (S2G Fig), with the total surface level of JAM-C expression increasing in a linear and highly correlative fashion for each JAM-C-EGFP concentration tested (S2H Fig). We therefore selected dilution rates of 1:1000 and 1:100 as representative dilutions, which increased JAM-C surface expression by 1.8-times (JAM-C-1.8x, level consistent with oxLDL stimulation [3]) and 6.6-times (JAM-C-6.6x) respectively.

We then evaluated whether JAM-C over-expression affected other key junctional molecules. We measured by FACS the expression level of VE-cadherin on normal HUVECs (JAM-C-WT) and transfected HUVECs (JAM-C-1.8x) (S2I Fig). No detectable difference was found. Furthermore, we also demonstrated that stimulating JAM-C-1.8x HUVECs with TNFα had no effect on JAM-C or VE-cadherin expression over 4-hrs (S2J Fig).

**References:**

1. Sircar M, Bradfield PF, Aurrand-Lions M, Fish RJ, Alcaide P, Yang L, et al. Neutrophil transmigration under shear flow conditions in vitro is junctional adhesion molecule-C independent. J Immunol. 2007;178(9):5879-87.

2. Lamagna C, Meda P, Mandicourt G, Brown J, Gilbert RJ, Jones EY, et al. Dual interaction of JAM-C with JAM-B and alpha(M)beta2 integrin: function in junctional complexes and leukocyte adhesion. Mol Biol Cell. 2005;16(10):4992-5003.

3. Keiper T, Al-Fakhri N, Chavakis E, Athanasopoulos AN, Isermann B, Herzog S, et al. The role of junctional adhesion molecule-C (JAM-C) in oxidized LDL-mediated leukocyte recruitment. FASEB J. 2005;19(14):2078-80.
