# Supplementary figures and images for: Divergent JAM-C Expression Accelerates Monocyte-Derived Cell Exit from Atherosclerotic Plaques
Source: PLoS One. 2016 Jul 21;11(7):e0159679. doi: 10.1371/journal.pone.0159679 (PMC4956249; doi:10.1371/journal.pone.0159679)

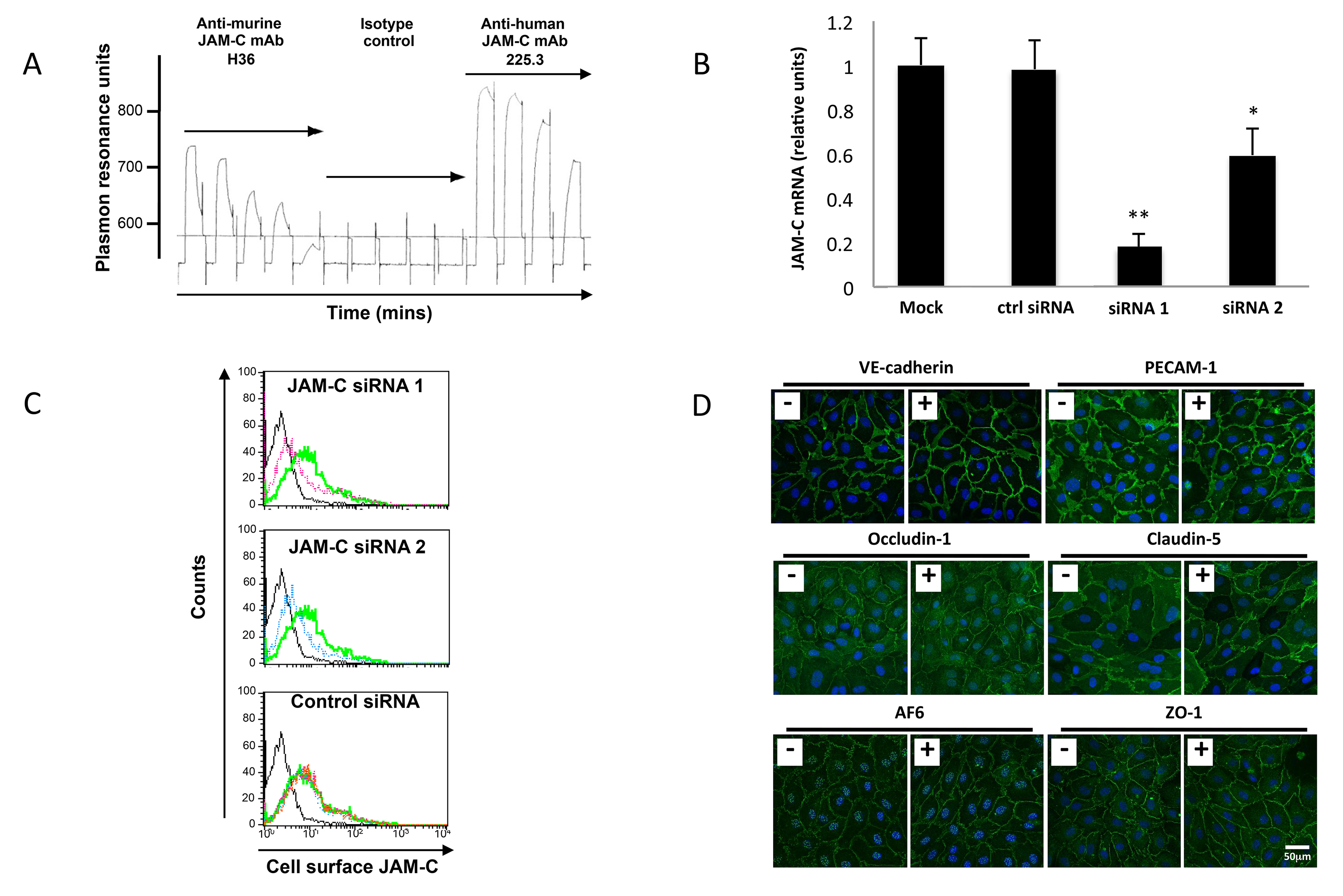

Supplement: S1 Fig — (A) Characterization of the novel anti-human JAM-C antibody 225.3. Biacore evaluation of anti-JAM-C Abs on immobilized soluble JAM-C comparing the anti-mouse JAM-C cross-reactive H36 Abs with the anti-human JAM-C 225.3. (B) Validation of JAM-C down-regulation on HUVECs after siRNA delivery by qPCR. Inhibition of JAM-C expression on cultured HUVECs after transfection with huJAM-C siRNA 1 and 2, when compared to control siRNA and mock control. All values were normalized to the expression levels of human beta-actin, beta-tubulin, and GAPDH. JAM-C siRNA 1 silenced > 75% of the huJAM-C mRNA in HUVECs after transfection and was used for all monocyte coculture experiments. Bars represent mean ± standard deviation (SD) (3 experiments, each condition in triplicate). P values were calculated compared to control siRNA (* = P<0.05; ** = P<0.01). (C) Validation of JAM-C down-regulation on HUVECs by flow cytometry. JAM-C expression on HUVECs was reduced after transfection with siRNA1 (pink) and 2 (blue), compared to JAM-C expression on non-transfected HUVECs (green). Expression of JAM-C on HUVECs transfected with control siRNA (blue) and non-transfected HUVECs remained comparable. An isotype control was included in all experiments (black). Histograms are representative of at least 2 experiments. (D) Overexpression of recombinant JAM-C does not affect distribution of other junctional proteins. Localization of the junctional proteins VE-Cadherin. PECAM-1. Occludin-1, Claudin-5, AF6 and ZO-1 were examined using confocal microscopy. No differences were observed between cells transfected with the control EGFP (marked as ‘-‘) and JAM-C-EGFP constructs (marked as ‘+‘). Images are representative of at least 4 independent experiments (N = 4). All antibody isotype controls included for immunofluorescence showed no staining (data not shown). (TIF) [file pone.0159679.s002.tif]

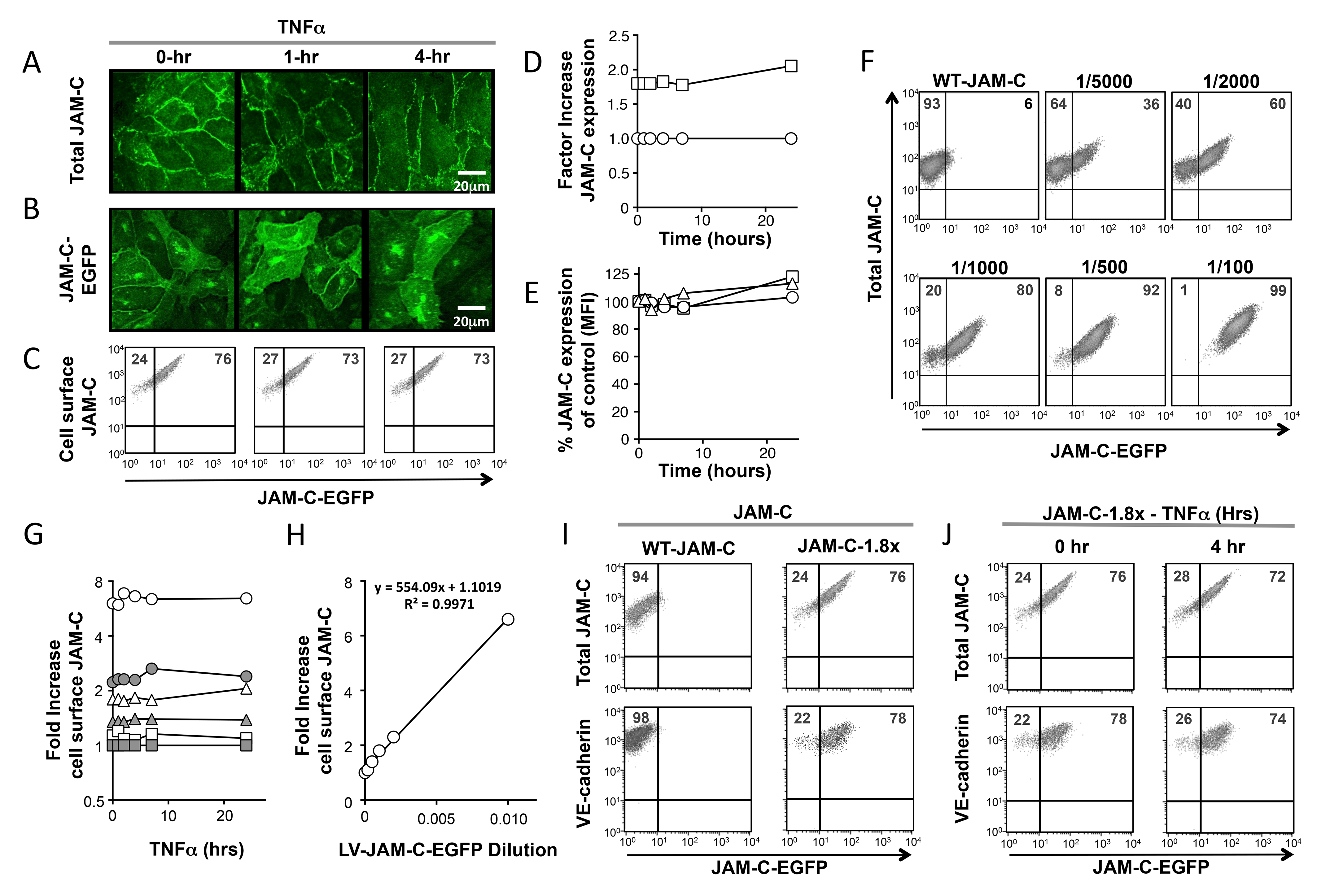

Supplement: S2 Fig — (A) Cultured HUVEC monolayers were stimulated with TNF-alpha and fixed at set time-points of 0 (unstimulated), 1- and 4-hrs. Human HUVECs were stained for human JAM-C using antibody 225.3 or an isotype control. JAM-C distribution remained unchanged throughout the 4-hr time-course with JAM-C remaining mostly in the junctions. The isotype control antibody showed no staining (data not shown). (B) Cultured HUVEC monolayers were transfected with JAM-C-EGFP lentivirus and stimulated with TNF-alpha at 0 (unstimulated), 1- and 4-hrs. Distribution of JAM-C-EGFP was similar to endogenous JAM-C and localized to intercellular junctions but also showed accumulation intracellularly. (C) Analysis by flow cytometry established total JAM-C expression in 73–76% of HUVECs transfected with JAM-C-EGFP and this increased in a linear fashion when compared to total JAM-C. Identical profiles were seen at 0-, 1- and 4-hrs after stimulation with TNF-alpha. (D) Increased total JAM-C expression using the JAM-C-EGFP construct (squares) was typically ~2-times higher than normal endogenous JAM-C expression (circles). (E) Comparison of JAM-C expression to the starting level of expression (MFI) in the endogenous (circles), total (squares) and JAM-C-EGFP populations (triangles) confirmed expression levels in each population were stable and remained unchanged up to 24-hrs. (F) An example set of flow cytometry profiles illustrating how total JAM-C expression increases with LV-JAM-C-EGFP load (G) Titration of LV-JAM-C-EGFP on cultured HUVECs. Flow cytometry studies indicated lentivirus JAM-C-EGFP preparation on cultured HUVECs stimulated with TNF-alpha increased total surface JAM-C expression in a dose-dependent manner. Titrations tested in this experiment were 1:100 (white circles), 1:500 (grey circles), 1:1000 (white triangle), 1:2000 (grey triangle), 1:5000 (white square) and a no virus control (grey square). Profiles of JAM-C expression remained constant at all concentrations up to 24-hrs for each c [file pone.0159679.s003.tif]

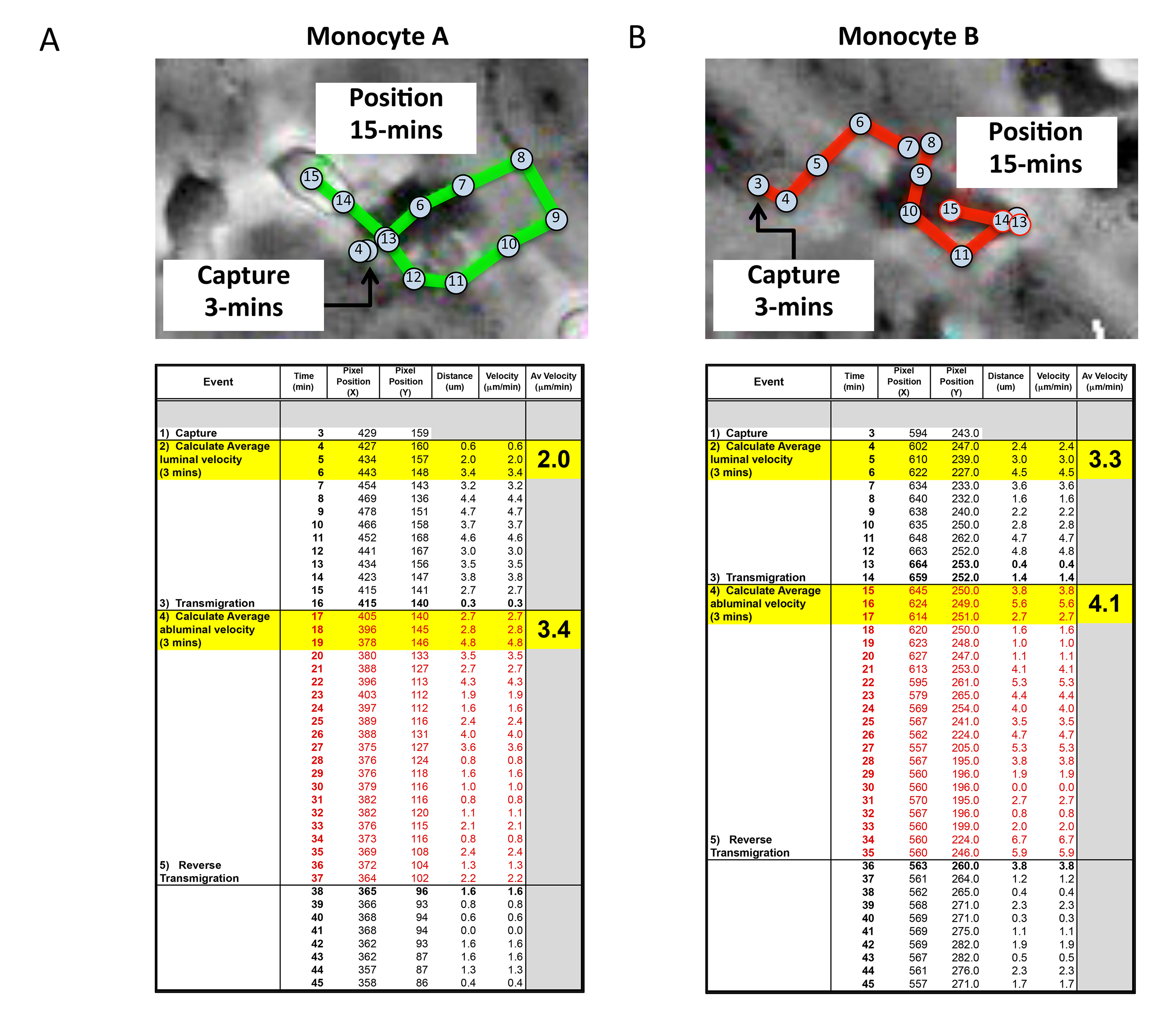

Supplement: S3 Fig — (A, B) Images are for two example monocytes (A and B) and contain representative cell tracking paths, plus a corresponding summary table detailing cell position and velocity analysis. The Capture point from flow, and cell position is represented by circles denoting the time (mins) and XY position. Circles with a black or red border denote a monocyte in the luminal and abluminal compartment respectively. The track direction is represented by green and red tracks for monocyte-A and -B respectively. An early timepoint of 15-mins was selected for the images in order to illustrate the tracking paths associated with each monocyte in different compartments. The extended track of Monocyte-A and–B can be observed as Monocyte-2 and -3 respectively in S2 Movie. The XY pixel coordinates and time for each individual monocyte were summarised in tables, allowing luminal and abluminal velocities to be recorded (data sets marked in black and red respectively). The average velocity for a given monocyte in each compartment was calculated over a 3-min period (highlighted in yellow). This was done with values immediately after monocyte capture (luminal velocity) or TEM (abluminal velocity). (TIF) [file pone.0159679.s004.tif]
